# Spatiotemporal Bayesian Regularization for Cardiac Strain Imaging: Simulation and *In Vivo* Results

**DOI:** 10.1109/OJUFFC.2021.3130021

**Published:** 2021-11-22

**Authors:** RASHID AL MUKADDIM, NIRVEDH H. MESHRAM, ASHLEY M. WEICHMANN, CAROL C. MITCHELL, TOMY VARGHESE

**Affiliations:** 1 Department of Medical Physics, University of Wisconsin School of Medicine and Public Health, Madison, WI 53706 USA; 2 Department of Electrical and Computer Engineering, University of Wisconsin–Madison, Madison, WI 53706 USA; 3 Small Animal Imaging and Radiotherapy Facility, UW Carbone Cancer Center, Madison, WI 53705 USA; 4 Department of Medicine/Division of Cardiovascular Medicine, University of Wisconsin School of Medicine and Public Health, Madison, WI 53792 USA

**Keywords:** Cardiac strain imaging, Bayesian regularization, spatiotemporal information, cardiac ultrasound, murine echocardiography, high frequency ultrasound, multi-level block matching

## Abstract

Cardiac strain imaging (CSI) plays a critical role in the detection of myocardial motion abnormalities. Displacement estimation is an important processing step to ensure the accuracy and precision of derived strain tensors. In this paper, we propose and implement Spatiotemporal Bayesian regularization (STBR) algorithms for two-dimensional (2-D) normalized cross-correlation (NCC) based multi-level block matching along with incorporation into a Lagrangian cardiac strain estimation framework. Assuming smooth temporal variation over a short span of time, the proposed STBR algorithm performs displacement estimation using at least four consecutive ultrasound radio-frequency (RF) frames by iteratively regularizing 2-D NCC matrices using information from a local spatiotemporal neighborhood in a Bayesian sense. Two STBR schemes are proposed to construct Bayesian likelihood functions termed as Spatial then Temporal Bayesian (STBR-1) and simultaneous Spatiotemporal Bayesian (STBR-2). Radial and longitudinal strain estimated from a finite-element-analysis (FEA) model of realistic canine myocardial deformation were utilized to quantify strain bias, normalized strain error and total temporal relative error (TTR). Statistical analysis with one-way analysis of variance (ANOVA) showed that all Bayesian regularization methods significantly outperform NCC with lower bias and errors (*p* < *0.001*). However, there was no significant difference among Bayesian methods. For example, mean longitudinal TTR for NCC, SBR, STBR-1 and STBR-2 were 25.41%, 9.27%, 10.38% and 10.13% respectively An *in vivo* feasibility study using RF data from ten healthy mice hearts were used to compare the elastographic signal-to-noise ratio (SNR_**e**_) calculated using stochastic analysis. STBR-2 had the highest expected SNR_**e**_ both for radial and longitudinal strain. The mean expected SNR_**e**_ values for accumulated radial strain for NCC, SBR, STBR-1 and STBR-2 were 5.03, 9.43, 9.42 and 10.58, respectively. Overall results suggest that STBR improves CSI *in vivo*.

## I. INTRODUCTION

CARDIAC strain imaging (CSI) estimates myocardial tissue elasticity by processing ultrasound (US) data corresponding to the natural contraction and relaxation of the myocardium [1], [2]. Applications of CSI in both clinical and preclinical domains (e.g. assessing myocardial ischemia in murine models [3], [4], monitoring cardiac radiofrequency ablation in human *in vivo* [5]) have been reported. Accurately estimating underlying cardiac motion or displacement is critical for CSI. The myocardium exhibits complex three-dimensional (3-D) motion patterns due to torsion, thickening and contraction along fibers, over a cardiac cycle [6]. This complex 3-D motion causes out-of-plane motion of scatterers when 2-D imaging is employed for CSI resulting in significant challenges for accurate strain quantification [7]. Incorporation of regularization (both in space and time) into cardiac strain estimation is an essential step and the main focus of this paper [8].

Displacement estimation algorithms for CSI can be broadly categorized into three classes: non-rigid image registration (NRIR), optimization and block matching (BM) with n-dimensional kernel-based (n = 1, 2 or 3) methods. Ledesma-Carbayo *et al*. [9] proposed a spatiotemporal elastic registration framework for estimating 2-D displacement fields by using a B-spline function-based parametric model representing the motion field. They enforced spatial smoothness and temporal coherence on the estimated deformation function by defining B-spline basis functions in both spatial and temporal directions to derive a globally plausible spatiotemporal motion field over the entire image sequence with respect to a reference frame (end-diastole). The approach was validated in a cardiac simulation model revealing the benefit of adding temporal consistency in the framework. A similar image intensity-based NRIR framework was applied to 3-D US image voxels by Elen *et al*. [10] to derive the cardiac motion field. Zhang *et al*. [11] proposed an elastic image registration framework for 3-D echocardiography images with spatiotemporal regularization (3-D + t approach). The temporal penalty term was defined where in three consecutive images any point in the myocardium will experience continuous velocity. Nora *et. al*. proposed spatial and sparse regularization with dictionary learning and reported better motion estimation accuracy compared to state-of-the-art methods [12], which was extended to incorporate temporal information [13]. Despite regularization being inherently embedded in these NRIR-based methods, they suffer from reduced sensitivity to small inter-frame displacements and lower elastographic signal-to-noise ratio (SNR_e_) due to the use of US B-mode or envelope data instead of RF data [14]. To address this issue, Bidisha *et al*. proposed a NRIR-based method for RF-based CSI [15]. However, their results did not include analysis on strain estimation accuracy limiting its effectiveness for CSI.

Optimization-based methods with RF-data generally minimize a regularized cost function with smoothness constraints incorporated [16]–[21]. For example, Hashemi *et al*. proposed Global US Elastography algorithm termed as GLUE where a non-linear optimization problem is formulated to estimate displacement in all RF A-lines simultaneously by enforcing a spatial constraint [17]. Rivaz *et al*. [22] applied the concept of temporal consistency in optimization-based displacement estimation using multiple RF frames. The proposed method initially estimates motion between paired images using 2-D analytic minimization (2-D AM) [20]. The initial estimates were then utilized to derive physics based constrains to construct a likelihood function to incorporate data from multiple images. Finally, a posterior probability density was constructed by combining the estimated likelihood function and spatial smoothening regularization term to derive final displacement estimates. The proposed method was compared against strain image averaging and Lagrangian particle tracking [23] and provided improved performance. Recently, Ashikuzzaman *et al*. proposed using the GLUE algorithm in spatial and temporal domain termed as GUEST to incorporate temporal continuity in the GLUE framework and validated their axial strain algorithm using simulation, phantom and *in vivo* liver data. However, these results are not generalizable to CSI where lateral and shear components play equally important roles in the derivation of cardiac strain tensors.

Deep learning-based motion tracking algorithms have also been utilized for ultrasound strain elastography [24]–[27]. Tehrani *et al*. proposed two convolutional neural networks based on pyramidal warping and cost volume network (PWC-Net) [28] by utilizing B-mode, envelope and RF data at different levels of the data pyramid for displacement estimation [24]. Ostvik *et al*. also modified PWC-Net for myocardial deformation imaging using clinical echocardiography data [29]. Delaunay *et al*. reported a recurrent neural network architecture with Long-Short-Term memory blocks to perform displacement estimation with spatiotemporal coherence [29]. Temporal coherence was modelled as a regularization term in the training loss to enforce temporal consistency between successive strain images [29]. Recently, Lu *et al*. proposed learning spatiotemporal regularization using a multi-layered perceptron neural network using biomechanical constraints during training [30].

Typically, RF-based CSI involves performing BM either with 2-D [23], [31] or 1-D kernels in a 2-D search region [32]–[37]. For BM displacement estimation algorithms, 1-D or 2-D kernels from pre-deformation RF data are matched with post-deformation kernels in a pre-defined search range using a similarity metric (e.g., sum of absolute difference, sum of squared difference, mutual information, phase correlation, normalized cross-correlation (NCC) [38]–[40]). Here, we focus on 2-D NCC based BM algorithm where the NCC peak location is used to obtain axial and lateral sub-sample shifts to determine the displacement vector. This approach is termed as **NCC** in the rest of the paper. We also denote 2-D NCC image as similarity metric image (SMI) for the ease of discussion.

Regularization can be included in BM algorithms either post estimation or during estimation. Examples of post estimation regularization include median filtering [32], [36], [41], application of geometric shape constraints on the estimated motion fields [42], Gaussian smoothening [43]. Examples of regularization during estimation include application of Viterbi algorithm [44]–[47] and Bayesian strain imaging [31], [48]–[51]. However, there are not many reports on the use of temporal consistency concept for kernel-based displacement estimation. Jiang *et al*. [52] proposed a method of estimating a composite strain image by processing multiple RF frames and then selecting three RF frames based on a displacement quality metric (DQM) [52]. A composite strain image was obtained by weighted averaging of the pair of strain images. Bayer *et al*. [53] explored temporal continuity based on the assumption that motion changes gradually over time. Recently, Mirzaei *et al*. proposed the use of 3-D NCC (2-D + time) and reported robustness against noise corruption for axial strain imaging [54]. Previously, we have demonstrated the use of Bayesian regularization in the context of multi-level BM-based CSI and reported significant performance improvement over conventional NCC without regularization [31], [55]–[57]. The proposed algorithm incorporated information from a local spatial neighborhood to regularize 2-D NCC matrices. Note that all previous reports on Bayesian strain imaging utilized information only from its spatial neighbors [31], [48]–[51], [55], [56], [58], [59]. Recently, we extended the Bayesian regularization algorithm into the temporal domain with the underlying assumption of smooth variation in velocity over a short span of time during tissue deformation [60] and performed limited validation studies using phantom and murine carotid RF data. However, an optimal scheme for temporal Bayesian regularization, performance variation with parametric sweeps along with detailed *in vivo* validation for cardiac applications were not investigated in the conference paper.

In this paper, we apply spatiotemporal Bayesian regularization (STBR) for CSI and perform a detailed feasibility study for cardiac application. The paper reports on two main contributions. First, two schemes for incorporating temporal domain information into our Bayesian regularization algorithm is proposed and implemented into a Lagrangian cardiac strain estimation framework [31]. Second, we report results from a comparative study involving conventional NCC, spatial and spatiotemporal Bayesian regularization using data from finite-element-analysis (FEA) canine cardiac simulation and ten healthy murine hearts collected *in vivo*.

## II. THEORETICAL BACKGROUND

### A. OVERVIEW OF BAYESIAN REGULARIZATION

McCormick *et al*. treated SMI as probability density function (PDF) images and used Bayes theorem for regularization by incorporating information from spatial neighboring BM locations [51]. A basic transformation [51] (addition of one and normalization of SMI values by their sum) was applied on the SMIs to obtain corresponding PDF images. The key idea behind Bayesian regularization is to develop a likelihood function, $\text{Pr}\left(u_{N_{x}}|u_{X}\right)$ with information from adjacent neighbors and calculate a posterior probability density (PPD), $\text{Pr}\left(u_{x}|u_{N_{x}}\right)$ using Bayes theorem as follows.

(1) $$\text{Pr}\left(u_{x}|u_{N_{x}}\right)=\frac{\text{Pr}\left(u_{N_{x}}|u_{x}\right)\text{Pr}\left(u_{x}\right)}{\text{Pr}\left(u_{N_{x}}\right)}$$

where, **u**_**x**_ is the displacement vector for the current BM location (**x**), $u_{N_{x}}$ is the set of displacement vectors from a spatial neighborhood, $N_{x}$ defined with four adjacent neighbors (left, right, top and bottom), Pr (**u**_**x**_) is the prior PDF corresponding to the SMI which is being regularized, and $\text{Pr}\left(u_{N_{x}}\right)$ is a normalization term. PPD denotes the regularized SMI after spatial information is combined with the unregularized prior PDF.

Assuming neighbors are independent to simplify mathematical modelling [55], the likelihood function $\text{Pr}\left(u_{N_{x}}|u_{X}\right)$ is defined as the multiplication of all neighboring PDFs, Pr (**u**_**x**′_|**u**_**x**_) which denotes the probability that a neighboring block at $x^{\prime }\in N_{x}$ has a displacement **u**_**x**′_ given displacement **u**_**x**_ at **x** and shown as below.

(2) $$\text{Pr}\left(u_{N_{x}}|u_{x}\right)=\prod_{x^{\prime }\in N_{x}}\text{Pr}\left(u_{x^{\prime }}|u_{x}\right)$$

The model used to define Pr (**u**_**x**′_|**u**_**x**_) is shown as follows.

(3) $$\text{Pr}\left(u_{x^{\prime }}|u_{x}\right)\propto \underset{v_{x^{\prime }}\in \text{D}}{\text{max}}\;\left[\text{Pr}\left(v_{x^{\prime }}\right)\times {\text{exp}}^{\left(\frac{-{\left\|v_{x^{\prime }}-u_{x}\right\|}^{2}}{2\sigma_{u}^{2}}\right)}\right]$$

Equation (3) indicates that for a possible displacement **u**_**x**′_, the Pr (**u**_**x**′_|**u**_**x**_) is defined as the maximum probability of a set of displacements (**v**_**x**′_ ∈ D) similar to **u**_**x**′_ weighted by a 2-D Gaussian term having the width for each direction defined a vector ***σ***_**u**_. The set of displacements, D is defined such that ∥**v**_**x**′_ − **u**_**x**_∥ < *ϵ* with *ϵ* = 3*σ*_*u*_. McCormick *et al*. coupled ***σ***_**u**_ with the maximum expected axial and lateral strain in an image by defining a parameter strain regularization sigma (SRS denoted by ***σ***_***ε***_). Finally, the regularized displacement estimator determines the integer displacement vector as the point where the PPD maximizes (equation 1) and achieves sub-sample precision through interpolation. This approach is termed as spatial Bayesian **(SBR)** displacement estimator in the rest of the paper. Note, this algorithm can be applied iteratively to incorporate information from non-adjacent neighbors.

### B. SPATIOTEMPORAL BAYESIAN REGULARIZATION (STBR)

**FIGURE 1.**
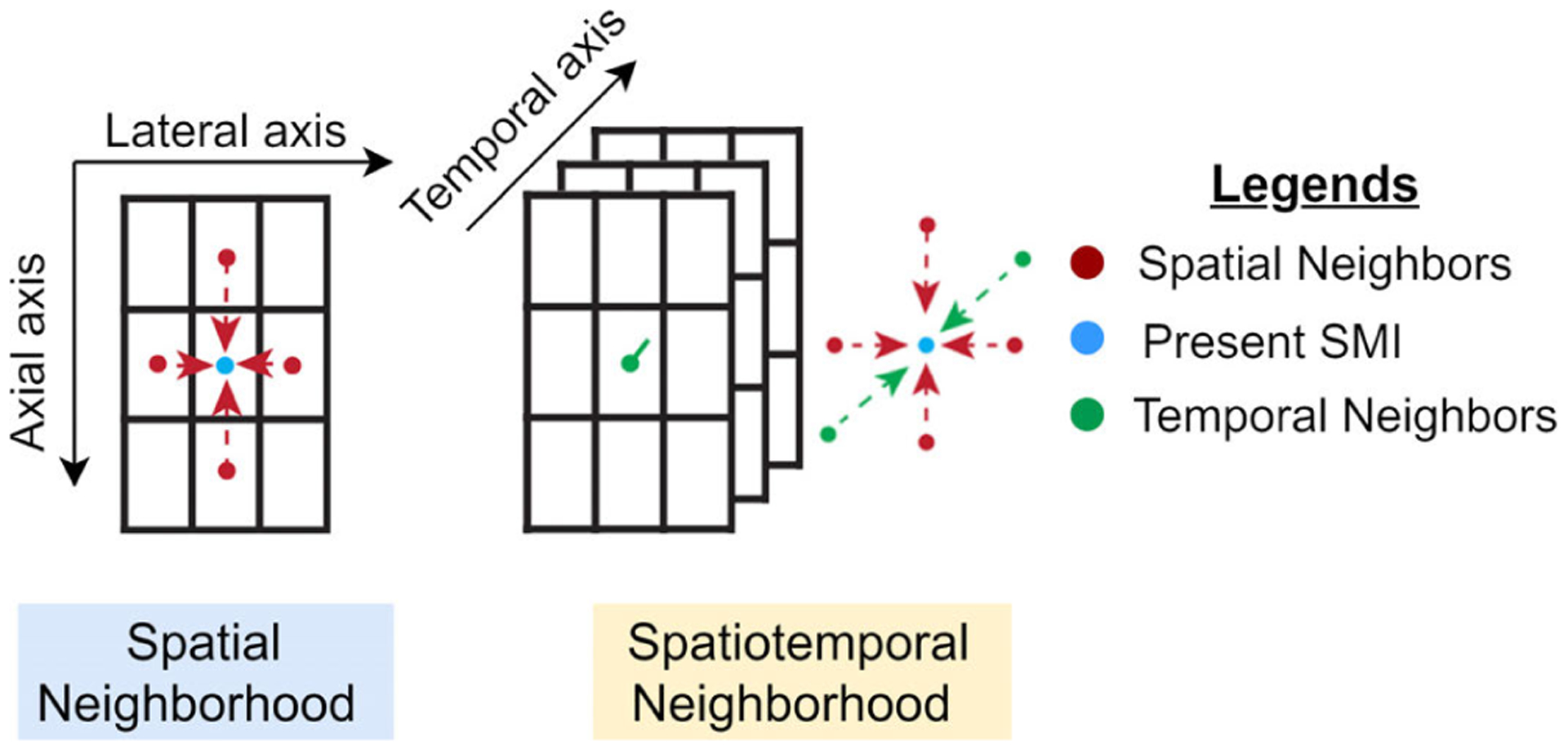
Neighborhood definition for spatial and spatiotemporal Bayesian regularization. The SMI being regularized is denoted by the blue circle while its spatial and temporal neighbors are indicated by red and green circles, respectively. Each rectangle represents a SMI.

For STBR, we consider a set of four consecutive RF frames for displacement estimation. Bayesian regularization is applied to SMIs; therefore, the regularization neighborhoods are defined in the SMI domain. As a pair of RF data frames result in a SMI at a BM location, we require four consecutive RF frames to have a minimum set of three SMIs at a BM location in the temporal domain. This is the smallest temporal neighborhood with past and future neighbors and is not linked to the acquisition frame rate and cardiac strain rate. However, if high frame rate acquisition is done or cardiac strain rate reduces, the proposed algorithm can be adapted to run with more than one iteration as the smooth variation in velocity assumption will hold for longer spans. First, inter-frame 2-D NCC estimation is performed resulting in three SMIs for each BM location. Specifically, for a BM location **x**, we have past, present and future temporal unregularized SMIs denoted by SMI(t − 1, **x**), SMI(t, **x**) and SMI(t + 1, **x**) respectively with SMI(t, **x**) being regularized by the proposed STBR method as shown in Figure 1. To enforce temporal continuity assuming smooth variation of velocity over time, we propose two schemes for incorporating temporal information into Bayesian regularization here as described below.

#### 1) SPATIAL THEN TEMPORAL BAYESIAN (STBR-1)

In this scheme, first one iteration of SBR is applied on all SMIs independently resulting into spatially regularized SMI for each BM location. Then, temporal regularization is done by considering these regularized SMI as the prior with a likelihood function incorporating information from its past and future temporal neighbors using following equation.

(4) $$\text{Pr}\left(u_{x}|u_{N_{t}}\right)\propto \text{Pr}\left(u_{N_{t}}|u_{x}\right)\times \text{Pr}\left(u_{x}|u_{N_{x}}\right)$$

where, $\text{Pr}\left(u_{X}|u_{N_{t}}\right)$ is the posterior PDF after temporal regularization, $u_{N_{t}}$ is the set of displacement vectors from a temporal neighborhood, $N_{t}$ defined with two adjacent neighbors (past and future) and $\text{Pr}\left(u_{X}|u_{N_{x}}\right)$ is PPD after one iteration of SBR. To define the temporal likelihood function $\left[\text{Pr}\left(u_{N_{t}}|u_{X}\right)\right]$, models like those reported in equations 2–3 are utilized and a 2-D temporal Gaussian term having the width vector ***σ***_**t**_ is defined. Finally, regularized displacement estimator determines the displacement vector as the point where $\text{Pr}\left(u_{x}|u_{N_{t}}\right)$ maximizes with sub-sample precision through interpolation. We term this method as **STBR-1** displacement estimation.

#### 2) SIMULTANEOUS SPATIOTEMPORAL BAYESIAN REGULARIZATION (STBR-2)

In the second scheme, STBR is done simultaneously on the present unregularized SMI using following equation.

(5) $$\text{Pr}\left(u_{X}|u_{N_{xt}}\right)\propto \text{Pr}\left(u_{N_{xt}}|u_{x}\right)\times \text{Pr}\left(u_{X}\right)$$

where, $\text{Pr}\left(u_{x}|u_{N_{xt}}\right)$ is the posterior PDF after spatiotemporal regularization, $u_{N_{xt}}$ is the set of displacement vectors from a spatiotemporal neighborhood, $N_{xt}$ defined with two adjacent temporal neighbors (past and future) and four adjacent spatial neighbors for the present SMI (left, right, top and bottom). To define the spatiotemporal likelihood function $\left[\text{Pr}\left(u_{N_{xt}}|u_{X}\right)\right]$, models like those reported in equations 2–3 are utilized with appropriate use of Gaussian terms for modulation depending on either spatial or temporal neighbors. Finally, *maximum a posteriori* (MAP) principle was applied on $\text{Pr}\left(u_{x}|u_{N_{xt}}\right)$ to determine displacement with sub-sample precision through interpolation. This approach is termed as **STBR-2** displacement estimator in this paper.

## III. MATERIALS AND METHODS

### A. CARDIAC FINITE-ELEMENT ANALYSIS SIMULATION STUDY

To evaluate the performance of STBR for CSI, a simulation study was performed using a 3-D FEA model of a healthy canine heart [61], [62] containing complex cardiac deformation over a cardiac cycle. Detailed description of FEA analysis and scatterer generation is described in [23], [31], [61]. Cardiac cycle RF data (125 frames) in 2-D parasternal long axis (PLAX) US imaging view extracted from the 3-D model was generated using a frequency domain US simulation program [63]. Transducer was modelled as a 1-D linear array having 128 elements (0.2 × 10 mm^2^) and a pitch of 0.2 mm operating at a center of frequency of 8.0 MHz and sampling frequency of 78.84 MHz. Simulated US images had a dimension of 80 × 100 mm^2^. US attenuation was modelled with attenuation co-efficient value set to 0.5-dB/cm-MHz. Sound of speed was assumed to be 1540 m/s for delay-and-sum beamforming. Five independent scatterer realizations were simulated for statistical analysis. For each scatterer realization, three sets of noisy RF datasets were generated by superimposing additive, white Gaussian noise (AWGN) on the simulated noise-less RF signals. AWGN profiles were generated relative to the noiseless RF signal derived from a 2-D region of interest (ROI) placed on the anterior wall. Furthermore, due to the simulated frequency dependent acoustic attenuation, the sonographic signal-to-noise (SNR_s_) of noisy RF data varied spatially over depth resulting in SNR_s_ = 45 dB, 15 dB and 7 dB respectively for anterior wall while SNR_s_ = 22 dB, 0 dB and −10 dB for posterior wall respectively [50]. Taking this SNR_s_ variation into consideration, we performed our simulation error analysis by dividing the cardiac wall into anterior and posterior segments rather than reporting the global error for the entire wall. Note that, for RF data with SNR_s_ < 0 dB all algorithms fail, and therefore were not included in the analysis.

### B. IN VIVO MURINE CARDIAC IMAGING

*In vivo* feasibility study was done by collecting cardiac RF data from 10 BALB/CJ mice (7 male, 3 female, median age = 10 weeks, acquired from Jackson Labs, Bar Harbor, ME, USA) using a Vevo 2100 system (FUJIFILM Visual-Sonics, Inc., Toronto, Canada). All *in-vivo* procedures were approved by the Institutional Animal Care and Use Committee (IACUC) at the University of Wisconsin-Madison. High frequency US imaging was performed using a MS 550D transducer (center frequency = 40 MHz). We acquired 1000 frames in PLAX view, which were stored in in-phase/quadrature (IQ) format for off-line CSI. Electrocardiogram (ECG) and respiratory signals were continuously monitored and simultaneously acquired during RF data collection. Finally, one cardiac cycle of RF data (sampling frequency = 512 MHz) was extracted from the collected 1000 frames by applying ECG and respiratory gating and used for CSI. The median heart rate of the mice scanned in the study was 293.40 beats per minute while the median number of frames in one cardiac cycle was 48. Further details regarding data collection can be found here [31].

### C. STBR ALGORITHM IMPLEMENTATION

**FIGURE 2.**
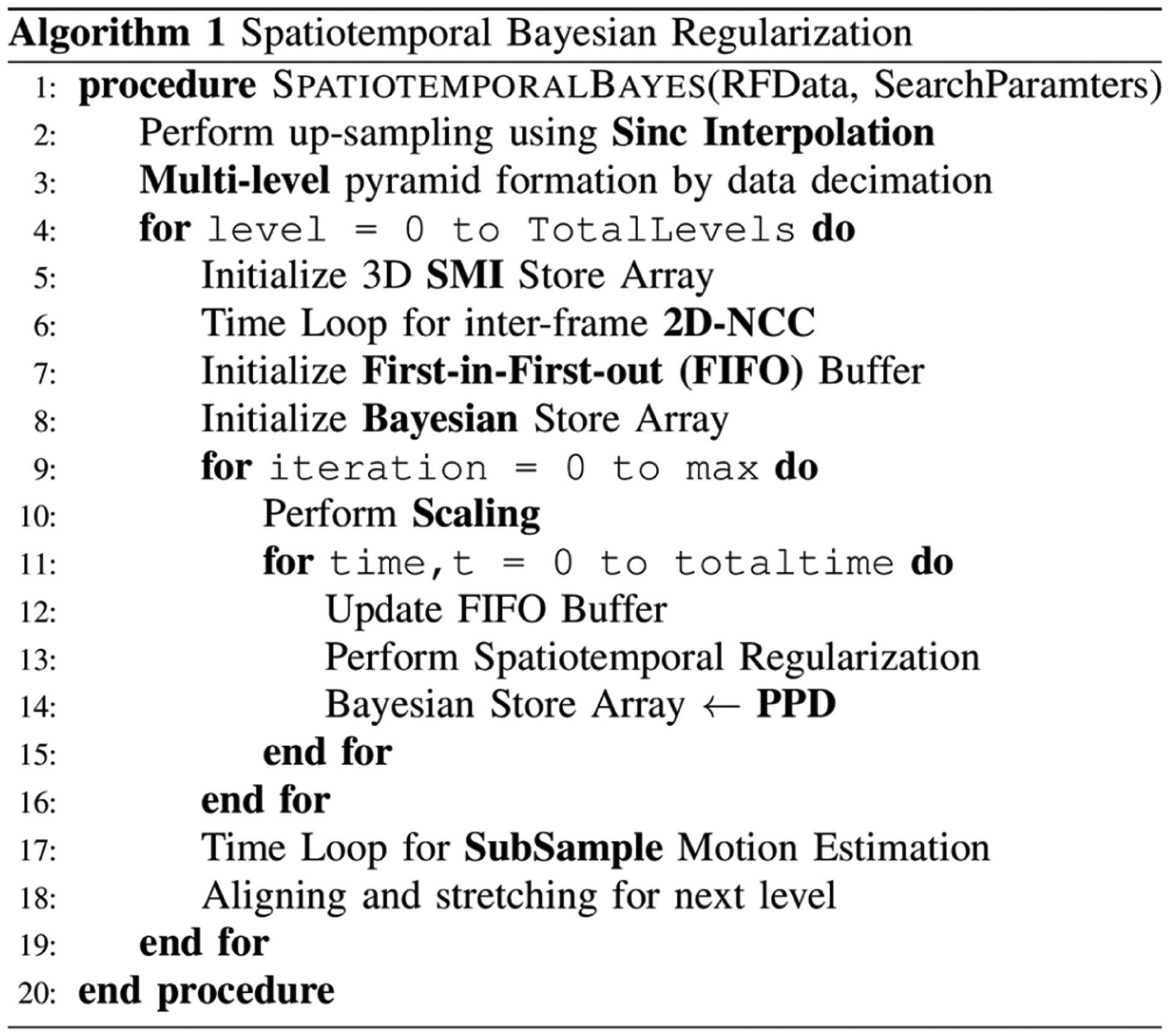
Proposed algorithm for STBR incorporated into a multi-level block matching displacement estimator. SMI = Similarity metric image, PPD = Posterior Probability Density.

STBR is incorporated into a multi-level BM algorithm [64] and implemented using MATLAB and CUDA to run on a GPU (NVIDIA Tesla K80) for cross-platform acceleration. Figure 2 presents pseudocode for the STBR algorithm where RFData and SearchParameters are structures containing four consecutive RF frames and displacement estimation parameters, respectively. The algorithm is as follows.

1. For all input frames, RF data are up-sampled using a 2-D windowed Sinc interpolator [57], [65] and multi-level pyramids are formed by data decimation.
2. At each level, inter-frame 2-D-NCC are estimated for all frames and stored in a 3-D SMI store array.
3. A First-in-First-out (FIFO) buffer and a 3-D Bayesian store array are initialized on GPU and CPU memory respectively for Bayesian regularization.
4. STBR is applied iteratively for all SMI using either equation 4 or 5. Perform Scaling in Figure 2 denote the normalization applied on SMIs to generate the PDFs. In this paper, we have limited STBR to a single iteration thus requiring only past and future neighbors for PPD calculation. However, to integrate information beyond adjacent temporal neighbors, we need more than four RF frames as an input to the algorithm resulting in higher memory requirement on the GPU. To avoid illegal memory access on GPU, the FIFO buffer holds required SMI data on GPU device memory for a specific time, t while results after performing regularization on GPU are copied back to the CPU Bayesian store array.
5. Finally, subsample motion estimation [57] is done with 2-D Sinc interpolation and RF data is prepared (by aligning and stretching [66]) for the next level.
6. Repeat steps (1) – (5) for the given number of levels.

### D. LAGRANGIAN CARDIAC STRAIN IMAGING

Lagrangian radial and longitudinal strain tensors were derived using a cardiac strain estimation framework proposed by our lab [31]. Inter-frame displacement estimation was performed with the multi-level BM algorithm [64] with and without regularization (SBR, STBR-1 and STBR-2). Note that, even though temporal information is utilized in the STBR method, the estimated displacement is still inter-frame. For example, if we have frames with ID 1, 2, 3 and 4 for STBR, estimated displacement would be between 2 and 3 and frames 1 and 4 were utilized in the Bayesian framework to incorporate temporal consistency. To ensure fair comparison among methods, frames 2 and 3 were also used as pre- and post-deformation frames for NCC and SBR-based displacement estimation. The displacement estimation parameters used for FEA simulation and *in vivo* studies are summarized in Table 1. Displacement estimation parameters for FEA simulation and *in vivo* studies were chosen based on the findings from our previous publications on Bayesian regularization and CSI [31], [55]. For STBR, width vector ***σ***_**t**_ was set empirically. Default axial and lateral direction *σ*_*t*_ values for FEA simulation and *in vivo* study were [0.01, 0.01] and [0.1, 0.1] respectively. A mesh of 24000 points covering the entire myocardium was generated by utilizing user-defined segmentation of epicardial and endocardial walls of the heart at end-diastole (ED) of a cardiac cycle (R-Wave of ECG) [23], [31]. The cardiac mesh was used then to integrate the inter-frame incremental displacements over time based on a Lagrangian description of motion starting from ED [31], [55], [56]. Before accumulation, 2-D median filtering was performed to remove any outliers from the estimated displacement vectors. The Lagrangian strain tensor (**E**) was derived by applying a least squares (LS) strain estimator on the accumulated displacement vectors to estimate axial, lateral and shear strain components [31], [33]. For FEA study, axial and lateral LS strain estimator kernel dimension was 0.5 mm and 1 mm respectively while *in vivo* study used axial and lateral kernels having dimension of 0.06 mm and 0.5 mm respectively to avoid smoothing/averaging during strain estimation. Finally, radial (*e*_*r*_) and longitudinal (*e*_*l*_) strains were derived by applying a coordinate transformation on **E** with details in [31]. End-systole (ES) strain images and segmental strain curves from both FEA simulation and *in vivo* mice data are used to qualitatively compare NCC, SBR, STBR-1 and STBR-2.

### E. QUANTITATIVE PERFORMANCE ANALYSIS

Theoretical strain tensors were derived from the 3-D cardiac FEA simulation and used to compare the strain estimation accuracy among NCC, SBR, STBR-1 and STBR-2 respectively. Quantitative performance analysis was done by evaluating the strain bias (%), normalized strain error (%) or Δ_*ε*_ (%) and total temporal relative error (TTR) as follows.

(6) $$\text{ Strain bias }(\%)=\text{E}\left[\varepsilon_{\text{true}\;}-\varepsilon_{\text{estimated}}\right]$$

(7) $$\Delta_{\varepsilon }(\%)=\frac{\sum_{i=1}^{P}\left|\varepsilon_{\text{true}\;}-\varepsilon_{\text{estimated}}\right|}{\sum_{i=1}^{P}\left|\varepsilon_{\text{true}}\right|}\times 100$$

(8) $$\text{TTR}(\%)=\frac{\sum_{t=1}^{T}\left|\varepsilon_{\text{true}}(t)-\varepsilon_{\text{estimated}}(t)\right|}{\sum_{t=1}^{T}\left|\varepsilon_{\text{true}}(t)\right|}\times 100$$

where, *ε*_*true*_ and *ε*_*estimated*_ denote estimated and theoretical strain images while *ε*_*true*_(*t*) and *ε*_*estimated*_ (*t*) denote the estimated and true strain value from segmental strain curves, respectively, *P* is the number of points in the cardiac mesh (24000 points) and *T* is the total number of frames in a cardiac cycle (125 frames). We computed strain bias and Δ_*ε*_ for each method at all-time points and for all scatterer realizations and concatenated the results in 1-D arrays for statistical analysis resulting in a sample size of 620 [6]. TTR quantified the resemblance between the true and estimated strain curves per scatterer realization resulting in a sample size of 5 [31]. One-way analysis of variance (ANOVA) with the Bonferroni multiple comparison test was done to determine statistical significance among NCC, SBR, STBR-1 and STBR-2. Statistical analysis was performed using MATLAB Statistics and Machine Learning Toolbox Version 11.4 (R2018b).

To compare the algorithm performance *in vivo*, strain filters [67] were derived for the accumulated radial and longitudinal strains at all time points for each method by performing stochastic precision analysis [67]–[69]. First, local elastographic signal-to-noise (SNR_*e*_) were computed as follows.

(9) $${\text{SNR}}_{\text{e}}=\frac{\mu }{\sigma }$$

where, *μ* and *σ* the mean and standard deviation of strain values within a 5 points × 9 points ROI centered at each cardiac mesh point. The window was translated over the entire cardiac mesh and calculation was repeated for all time points within a cardiac cycle resulting into strain-SNR_*e*_ pairs which were used to generate a 2-D histogram representing the SNR_*e*_ PDF, *f* (SNR_e_, *ε*) and a 1-D histogram representing the strain PDF, *f* (*ε*). Then, *f* (SNR_e_, *ε*) was normalized by *f* (*ε*) resulting into the conditional PDF, *f* (SNR_e_, *ε*). Finally, strain filter or the conditional expected value of the SNR_*e*_ was derived using the follow equation.

(10) $$E\left({\text{SNR}}_{\text{e}}|\varepsilon \right)=\int_{0}^{+\infty }{\text{SNR}}_{\text{e}}\times f\left({\text{SNR}}_{\text{e}}|\varepsilon \right)d{\text{SNR}}_{\text{e}}$$

To perform comparative analysis among NCC, SBR, STBR-1 and STBR-2, we qualitatively compared the corresponding strain filters. Additionally, *E*(SNR_e_ |*ε*) values for radial and longitudinal strains at 46% and −17.69% strains were compared by one-way analysis of variance (ANOVA) with the Bonferroni multiple comparison test following an approach reported in [35].

## IV. RESULTS

### A. CARDIAC FEA SIMULATION STUDY

**FIGURE 3.**
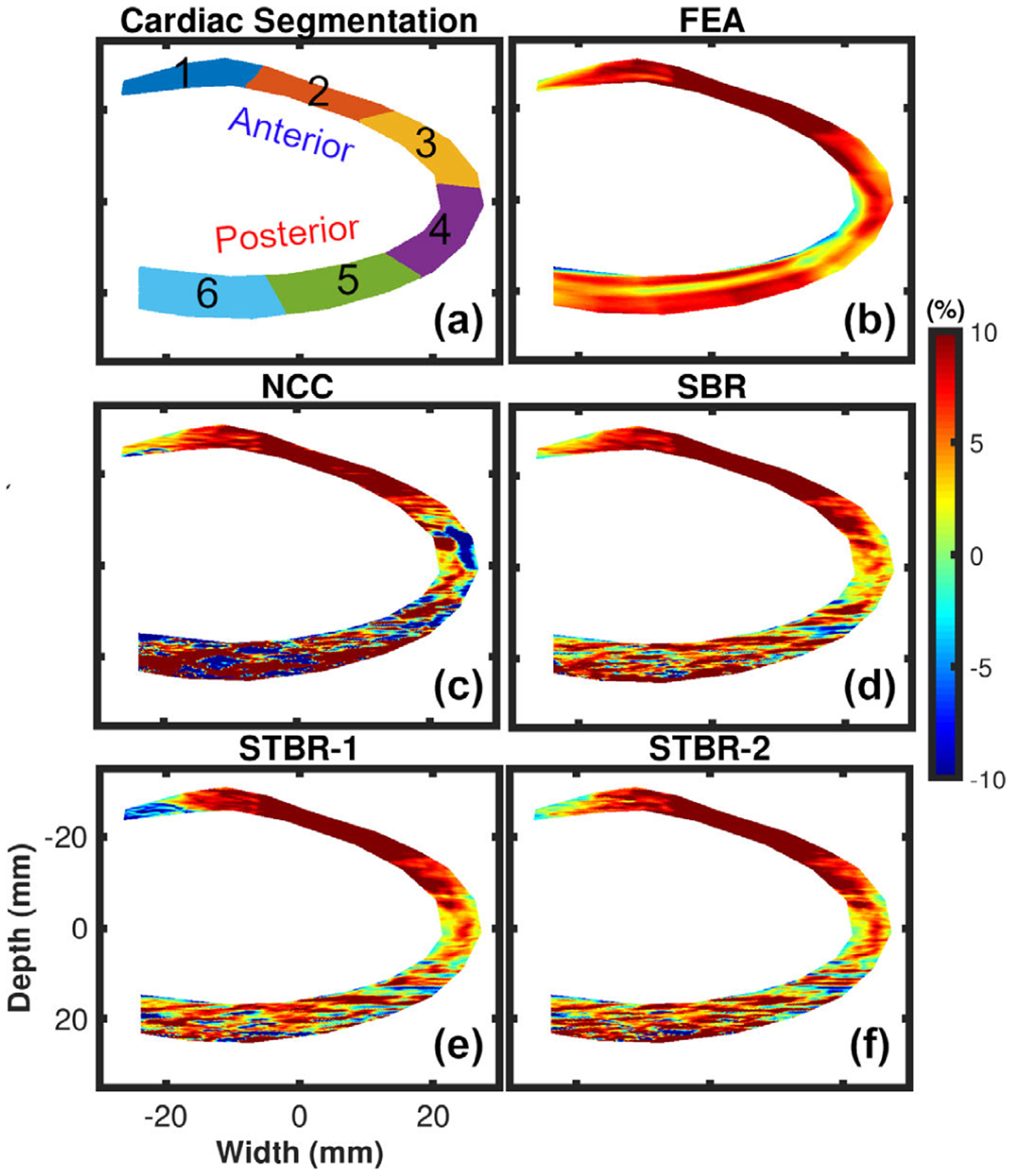
Qualitative comparison of ES radial strain estimation for FEA simulations. (a) Cardiac segments used for regional analysis. (b) – (f) denote FEA, NCC, SBR, STBR-1 and STBR-2 results, respectively. SNR_s_ values at anterior and posterior wall = 15 dB and 0 dB respectively.

Figures 3 (b) – (f) show ES radial strain images obtained using FEA model, NCC, SBR, STBR-1 and STBR-2, respectively. Input RF data for this example had SNR_s_ value of 15 dB at anterior wall and 0 dB at posterior wall. Radial thickening of myocardium at ES was observed in the FEA result with positive strain values. The myocardium was divided into six equal segments denoted as segments 1 – 6 respectively in Figure 3 (a). Segments 1 – 6 denote anterior base, anterior mid, anterior apex, posterior apex, posterior mid and posterior base segments respectively. NCC had noisy estimates in apical and posterior segments (3 – 6) with spuriously elevated positive and negative strain values. Regularization (SBR, STBR-1 and STBR-2) reduced strain noise when compared to NCC in segments 3 – 6. STBR-1 suffered from underestimation in anterior base (segment 1).

**FIGURE 4.**
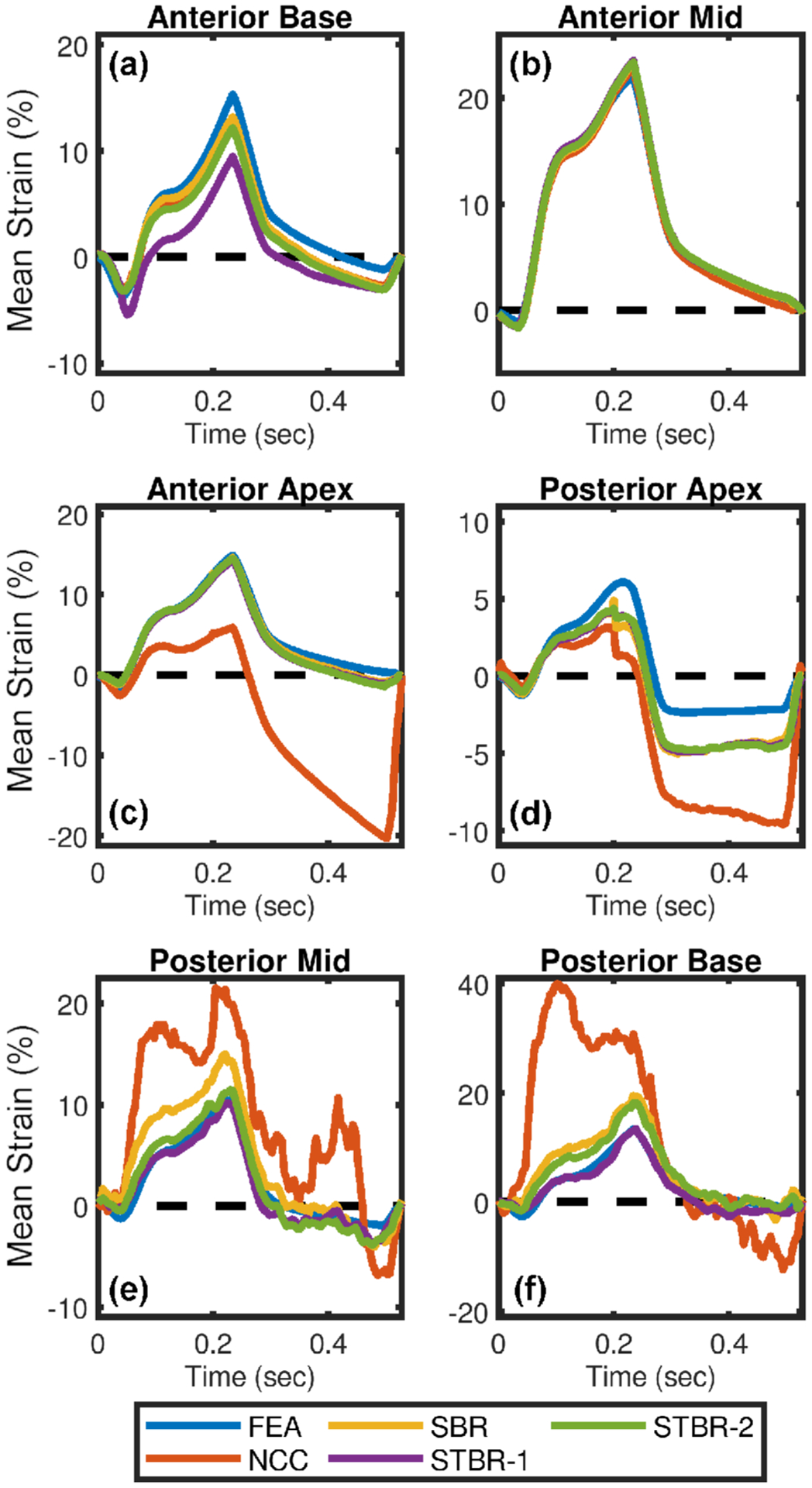
Qualitative comparison of radial strain curves for FEA simulation. Radial strain curves comparison among NCC, SBR, STBR-1 and STBR-2 for (a) anterior base, (b) anterior mid, (c) anterior apex, (d) posterior apex, (e) posterior mid and (f) posterior base segments respectively. SNR_s_ values at anterior and posterior wall = 15 dB and 0 dB respectively.

Segmental radial strain curves corresponding to Figure 3 are summarized in Figure 4. Figures 4 (a) – (f) compare the segmental radial strain curves estimated using NCC, SBR, STBR-1 and STBR-2 for anterior base, anterior mid, anterior apex, posterior apex, posterior mid and posterior base segments respectively against FEA results. NCC results had higher deviation from the FEA in apical and posterior segments (Figures 4 (c) – (f)). Significant improvement in strain estimation quality was achieved with SBR, STBR-1 and STBR-2 methods. STBR improved the quality further in posterior mid and posterior base segments compared to SBR (observe the STBR-2 results in Figure 4 (e)). However, STBR-1 underestimated radial strain in anterior base segment corroborating the finding from Figure 3.

**FIGURE 5.**
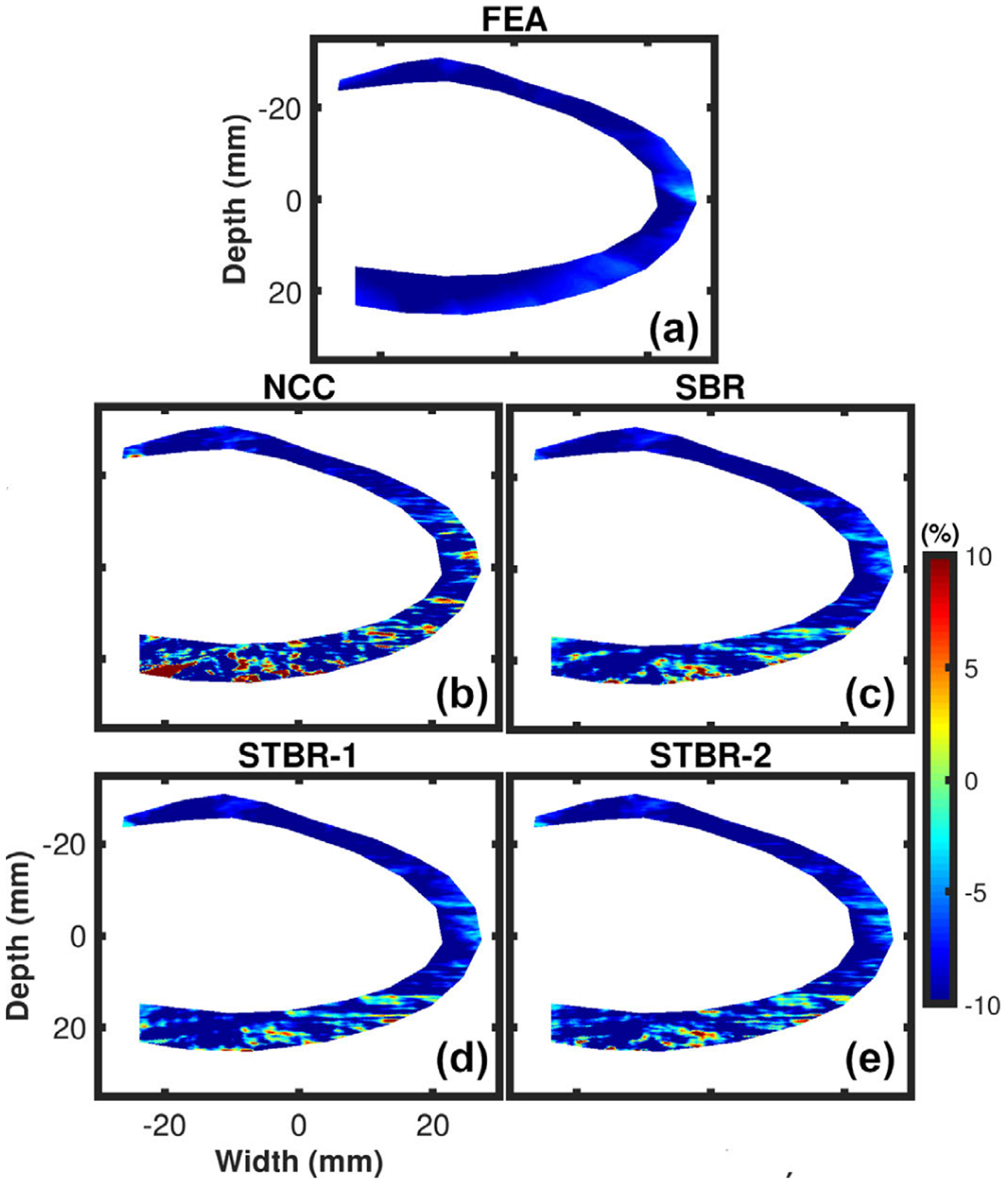
Qualitative comparison of ES longitudinal strain estimation for FEA simulation. (a) – (e) denote FEA, NCC, SBR, STBR-1 and STBR-2 results, respectively. SNR_s_ values at anterior and posterior wall = 15 dB and 0 dB respectively.

Figures 5 (a) – (e) show ES longitudinal strain images obtained using FEA, NCC, SBR, STBR-1 and STBR-2, respectively. Longitudinal shortening of myocardium at ES was observed in the FEA result with uniform negative strain values. NCC had noisy estimates in apical and posterior segments (3 – 6) with spurious high positive negative strain values. All regularization methods (SBR, STBR-1 and STBR-2) reduced strain noise compared to NCC in segments 3 – 6 with better qualitative agreement with FEA. No significant qualitative difference was observed among SBR, STBR-1 and STBR-2 results.

**FIGURE 6.**
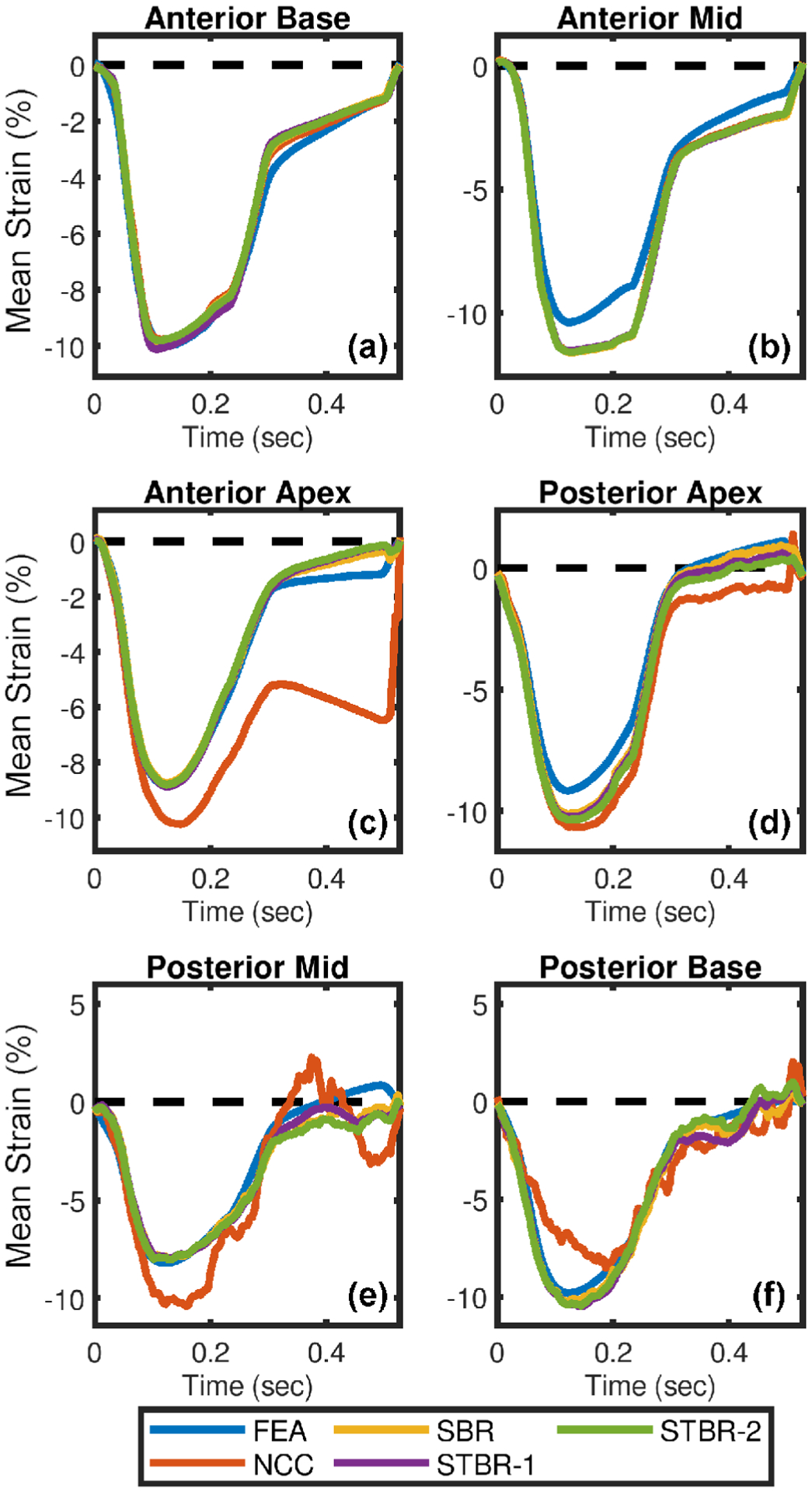
Qualitative comparison of longitudinal strain curves for FEA simulation. Longitudinal strain curves comparison among NCC, SBR, STBR-1 and STBR-2 for (a) anterior base, (b) anterior mid, (c) anterior apex, (d) posterior apex, (e) posterior mid and (f) posterior base segments respectively. SNR_s_ values at anterior and posterior wall = 15 dB and 0 dB respectively.

Comparison of segmental longitudinal strain curves shown in Figure 5 are summarized in Figure 6. Figures 6 (a) – (f) compare NCC, SBR, STBR-1 and STBR-2 for the 6 segments versus FEA results. NCC results had higher deviation from the FEA in apical and posterior segments (Figures 6 (c) – (f)). Significant improvement in strain estimation quality was achieved with SBR, STBR-1 and STBR-2 methods compared to NCC with no significant difference among each other.

**FIGURE 7.**
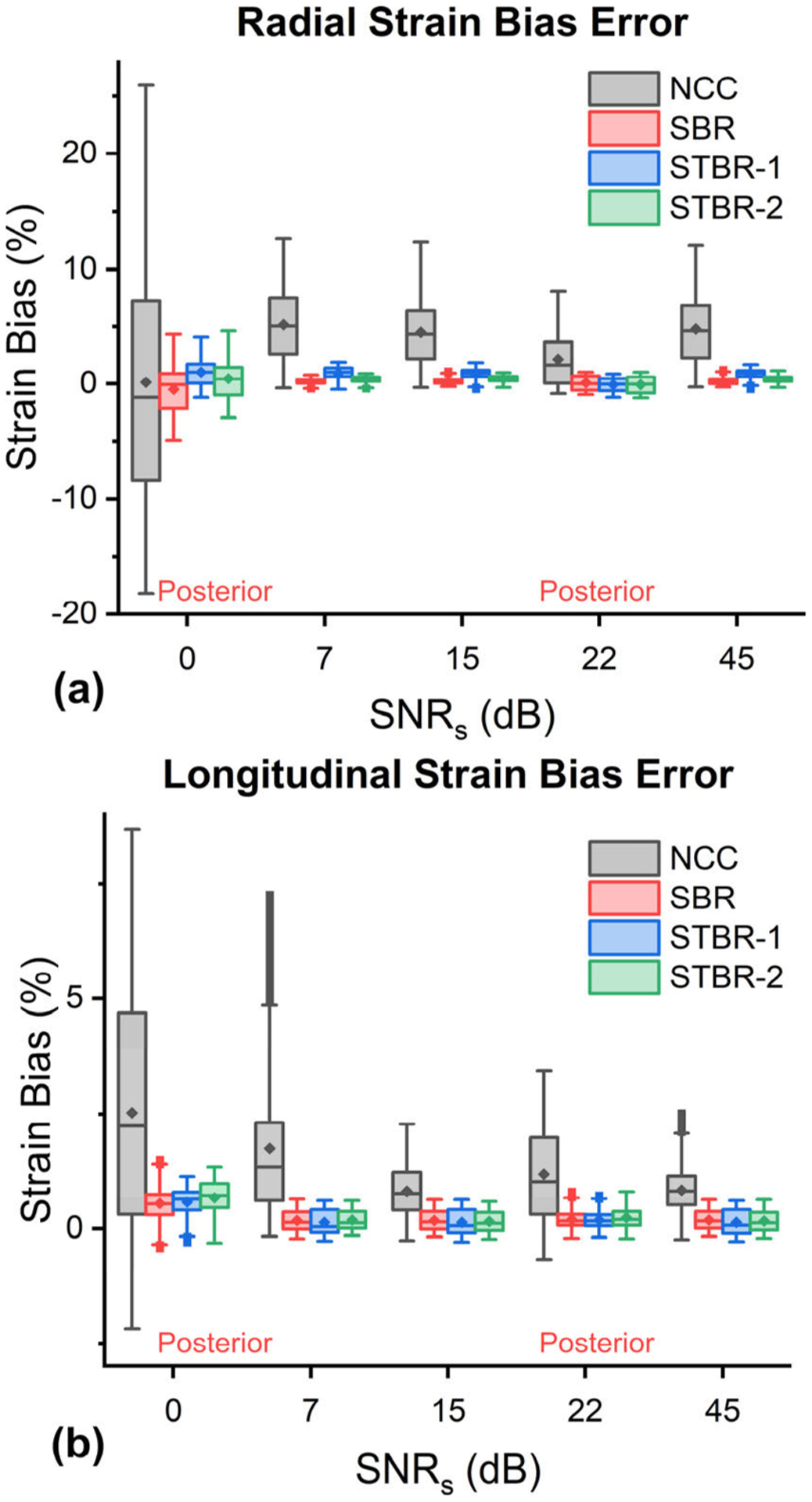
Strain estimation bias comparison (n = 620). (a) – (b) Radial and longitudinal strain estimation bias as a function of SNR_s_ levels sampled from anterior and posterior walls. SNR_s_ = 0 and 22 dB were from posterior wall.

Figure 7 summarizes the comparison results for strain estimation bias. Figures 7 (a) – (b) show radial and longitudinal strain estimation bias for anterior and posterior cardiac segments as a function of input RF data SNR levels. Both SBR and STBR methods had lower radial strain estimation bias with statistical significance (*p* < *0.001*). However, in some cases, STBR-1 had higher radial strain bias compared to SBR and STBR-2 with statistical significance. All regularization methods had lower longitudinal strain estimation bias with statistical significance (*p* < *0.001*) compared to NCC with no statistically significant difference among each other. For example, for 22 dB data at posterior wall, mean *e*_*l*_ estimation bias for NCC, SBR, STBR-1 and STBR-2 were 1.20%, 0.19%, 0.19% and 0.23% respectively.

**FIGURE 8.**
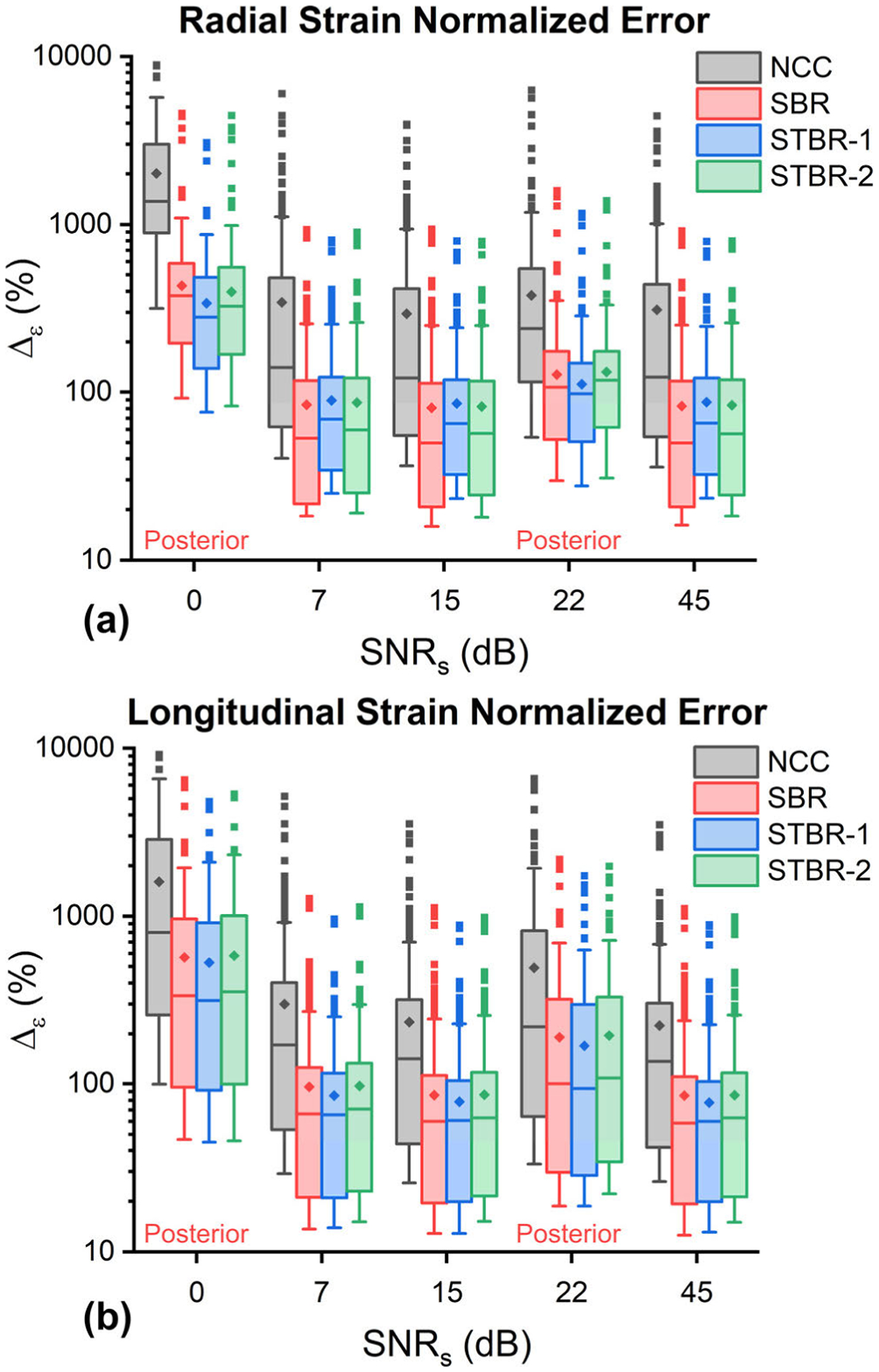
Normalized strain error or Δ_*ε*_(%) comparison (n = 620). (a) – (b) Radial and longitudinal Δ_*ε*_(%) as a function of SNR_s_ levels sampled from anterior and posterior walls. SNR_s_ = 0 and 22 dB were from posterior wall.

Figure 8 summarizes the comparison results for normalized strain error or Δ_*ε*_(%). Radial strain and longitudinal Δ_*ε*_(%) for anterior and posterior cardiac segments as a function of RF data SNR_s_ are presented in Figures 8 (a) – (b) respectively. All regularization methods performed significantly better than NCC (*p* < *0.001*) with no statistically significant difference among each other. All methods demonstrated higher normalized strain error as the noise level of RF data increased.

**FIGURE 9.**
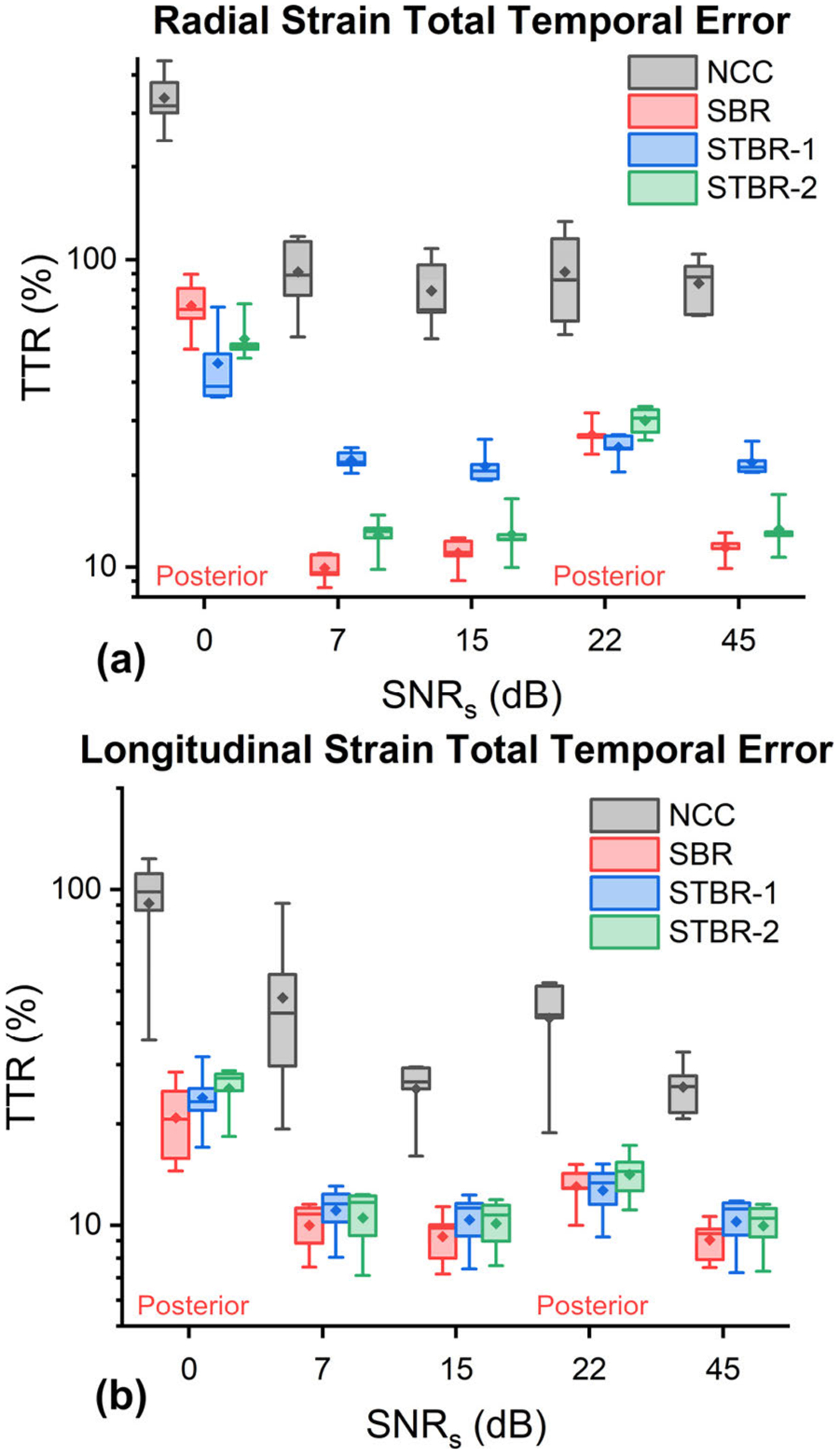
TTR error comparison (n = 5). (a) – (b) Radial and longitudinal TTR as a function of SNR_s_ levels sampled from anterior and posterior walls. SNR_s_ = 0 and 22 dB were from posterior wall.

Figures 9 (a) – (b) show radial and longitudinal TTRs as a function of RF data SNR_s_ respectively. All regularization methods performed significantly better than NCC for both radial and longitudinal strain. For posterior wall (SNR_s_ = 0 and 22 dB), STBR-1 had lower radial TTR compared to SBR and STBR-2. However, at the anterior wall, STBR-1 had significantly higher radial TTR compared to SBR and STBR-2 thus balancing out the performance improvement in the posterior wall. SBR had lower longitudinal TTRs compared to STBR methods. However, the values did not differ significantly. (For example, at SNR_s_ = 15 dB, mean *e*_*l*_ TTR for NCC, SBR, STBR-1 and STBR-2 were 25.41%, 9.27%, 10.38% and 10.13% respectively.

**FIGURE 10.**
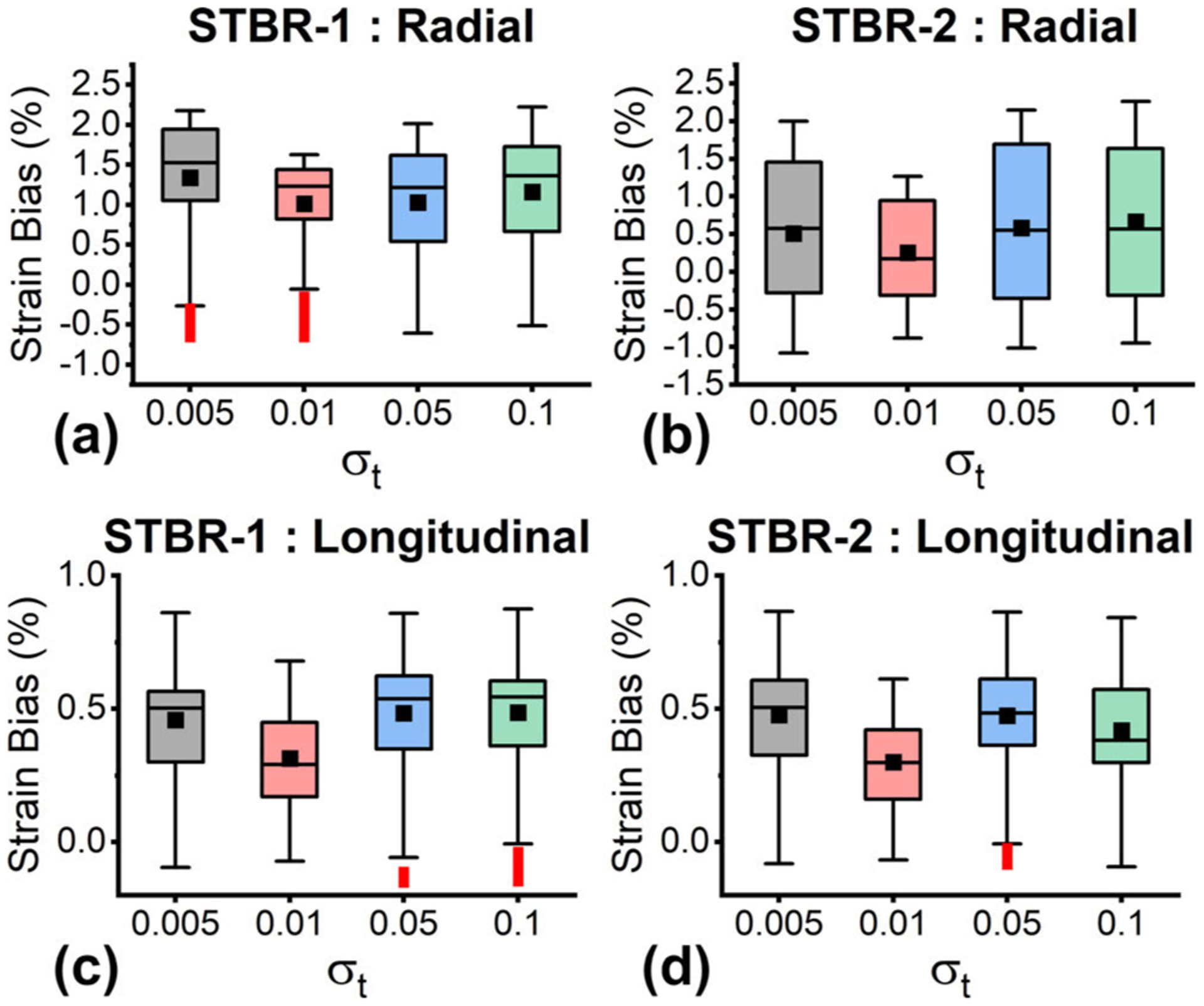
Variation of strain estimation bias as a function of *σ*_*t*_ (n = 125). (a) – (b) Variation of radial strain estimation bias as a function of *σ*_*t*_ for STBR-1 and STBR-2 respectively. (b) Variation of longitudinal strain estimation bias as a function of *σ*_*t*_ for STBR-1 and STBR-2 respectively.

Figure 10 shows the variation of strain estimation bias as a function of width vector ***σ***_**t**_. Figures 10 (a) – (b) show the variation of radial strain estimation bias for STBR-1 and STBR-2 respectively while Figures 10 (c) – (d) show the variation of longitudinal strain estimation bias. Width vector = [0.01, 0.01] had the lowest bias for all cases and is therefore used as a default parameter in the FEA study.

### B. IN VIVO MURINE CARDIAC IMAGING

**FIGURE 11.**
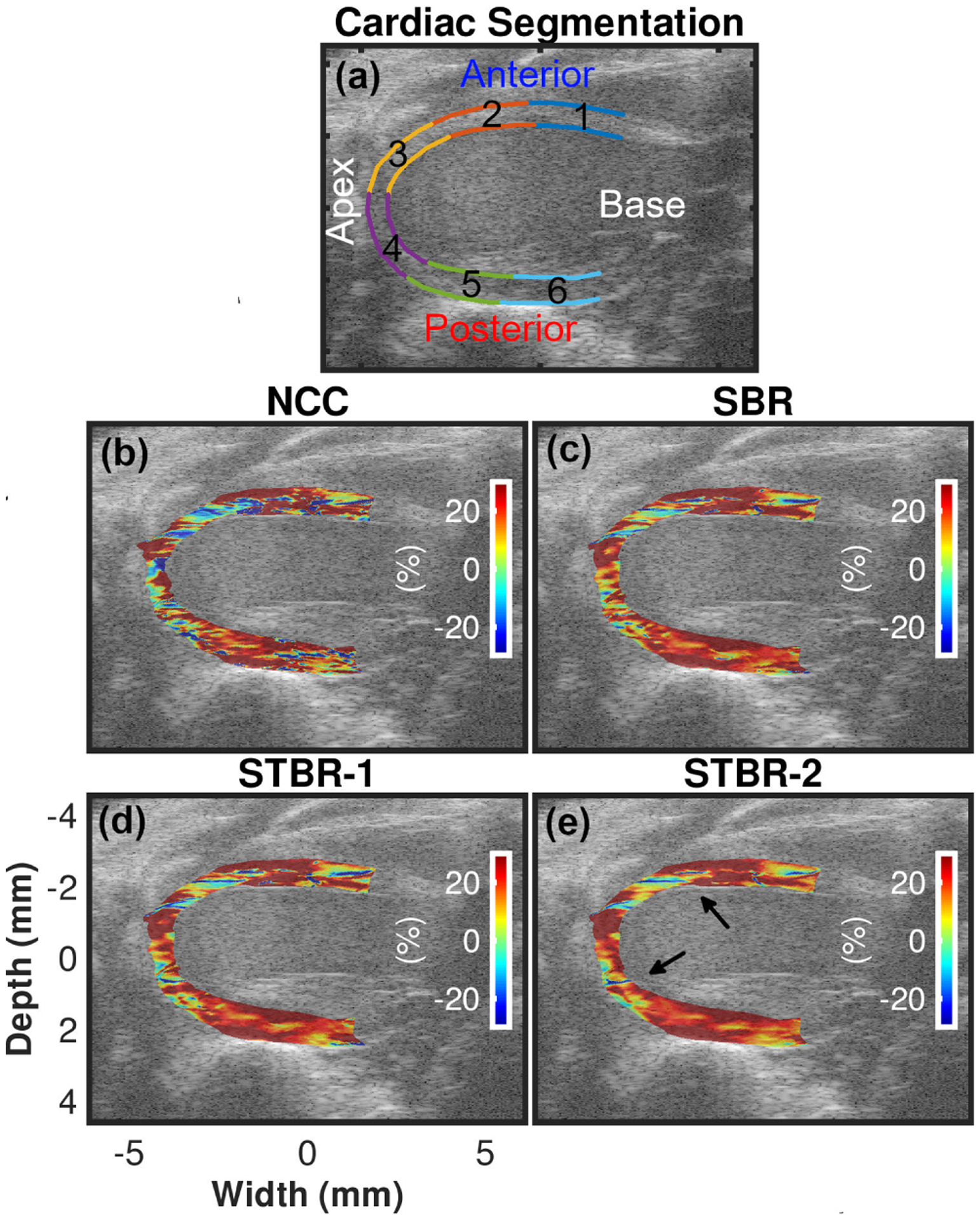
*In vivo* ES radial strain image comparison. (b) – (e) Radial strain images estimated with NCC, SBR, STBR-1 and STBR-2 respectively. Segments 1–6 shown in Fig. 11 (a) denote anterior base, anterior mid, anterior apex, posterior apex, posterior mid and posterior base segments respectively.

Figures 11 (b) – (e) show ES radial strain images obtained using NCC, SBR, STBR-1 and STBR-2, respectively for a healthy mouse heart. Segments 1–6 shown in Figure 11 (a) denote anterior base, anterior mid, anterior apex, posterior apex, posterior mid and posterior base segments respectively used for segmental analysis. Radial thickening of myocardium at ES was observed in all results. However, NCC depicts patches of spuriously high non-physiological negative strain values throughout the entire myocardium. All regularization methods significantly reduced these erroneous strain values providing performance improvement. The best strain distribution was achieved with STBR-2 *in vivo* (observed regions indicated with arrows) correlating with the physiological expectation from a healthy mouse heart.

**FIGURE 12.**
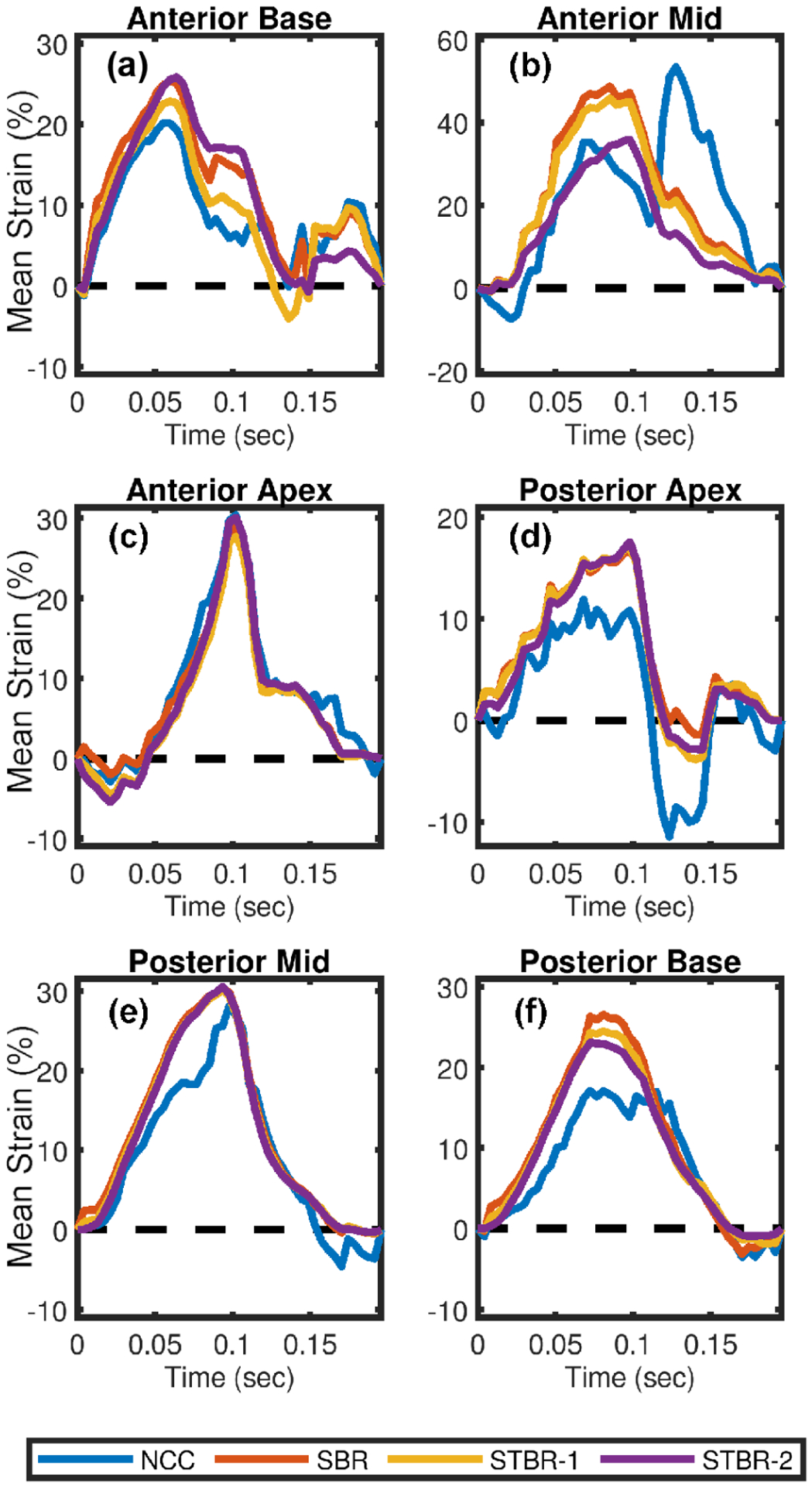
*In vivo* qualitative comparison of radial strain curves. Radial strain curves comparison among NCC, SBR, STBR-1 and STBR-2 for (a) anterior base, (b) anterior mid, (c) anterior apex, (d) posterior apex, (e) posterior mid and (f) posterior base segments respectively.

Figures 12 (a) – (f) compare segmental radial strain curves estimated using NCC, SBR, STBR-1 and STBR-2 for the 6 segments [shown in Figure 11 (a)] respectively. NCC without regularization resulted in noisy radial strain curves. For example, observe the peak shift and temporal jitter noise in anterior mid and posterior apex segments respectively. Significantly better radial strain curves were obtained using Bayesian regularization (both spatial and spatiotemporal). STBR-2 had the best quality curves quantified in terms of physiological relevant strain variation and temporal smoothness thus corroborating the ES strain image quality observation from Figure 11.

**FIGURE 13.**
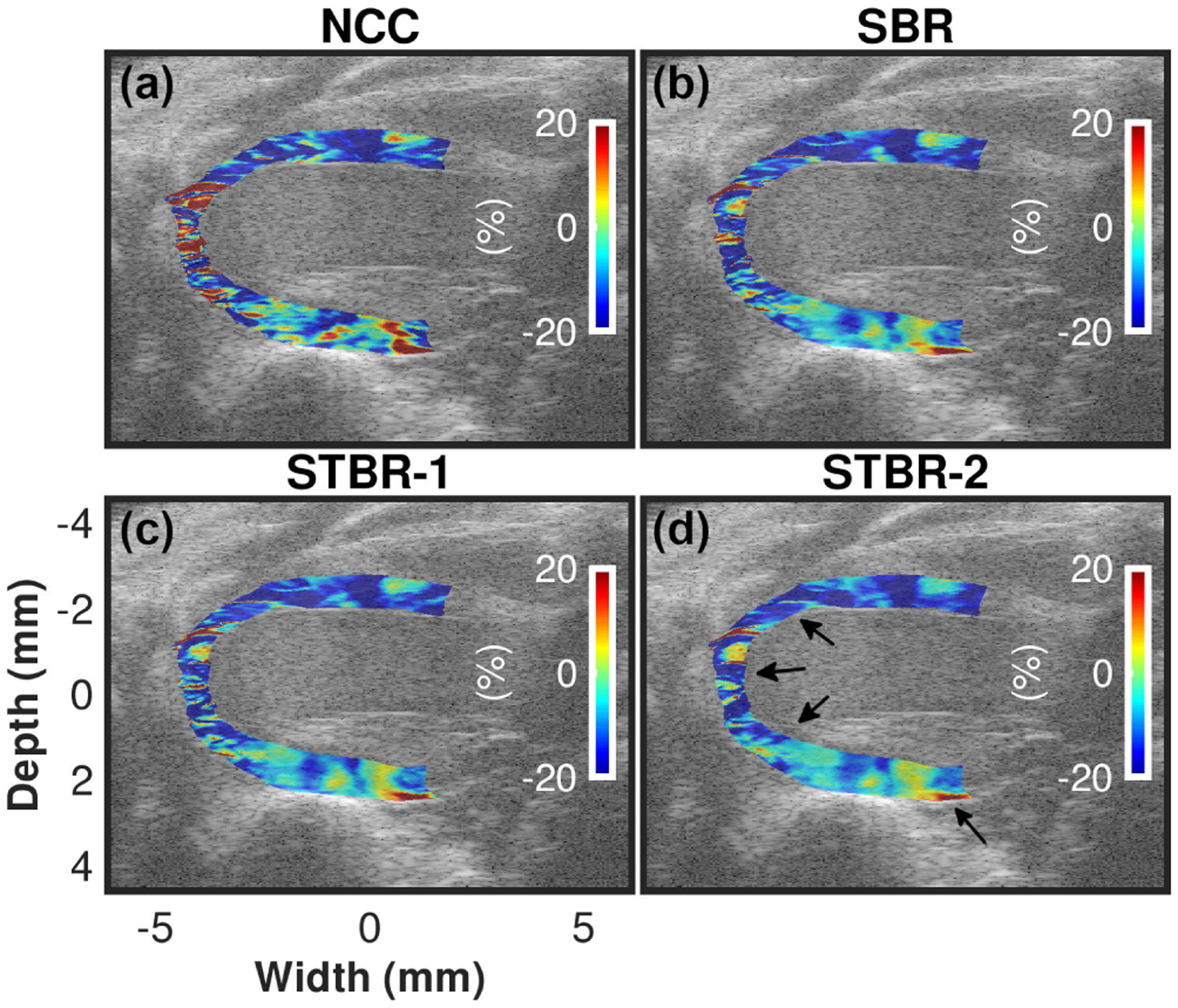
*In vivo* ES longitudinal strain image comparison. (a) – (d) Longitudinal strain images estimated with NCC, SBR, STBR-1 and STBR-2 respectively.

Figures 13 (a) – (d) show ES longitudinal strain images obtained using NCC, SBR, STBR-1 and STBR-2, respectively for a healthy mouse heart. Longitudinal shortening of myocardium at ES was observed in all results. However, NCC depict patches of spuriously high unphysiological positive strain values throughout the entire myocardium with higher concentration in the apical and posterior base segments. All regularization methods significantly reduced those erroneous strain values providing performance improvement. The most homogeneous strain distribution was achieved with STBR-2 *in vivo* with significant improvement in the apical regions (observed regions indicated with arrows).

**FIGURE 14.**
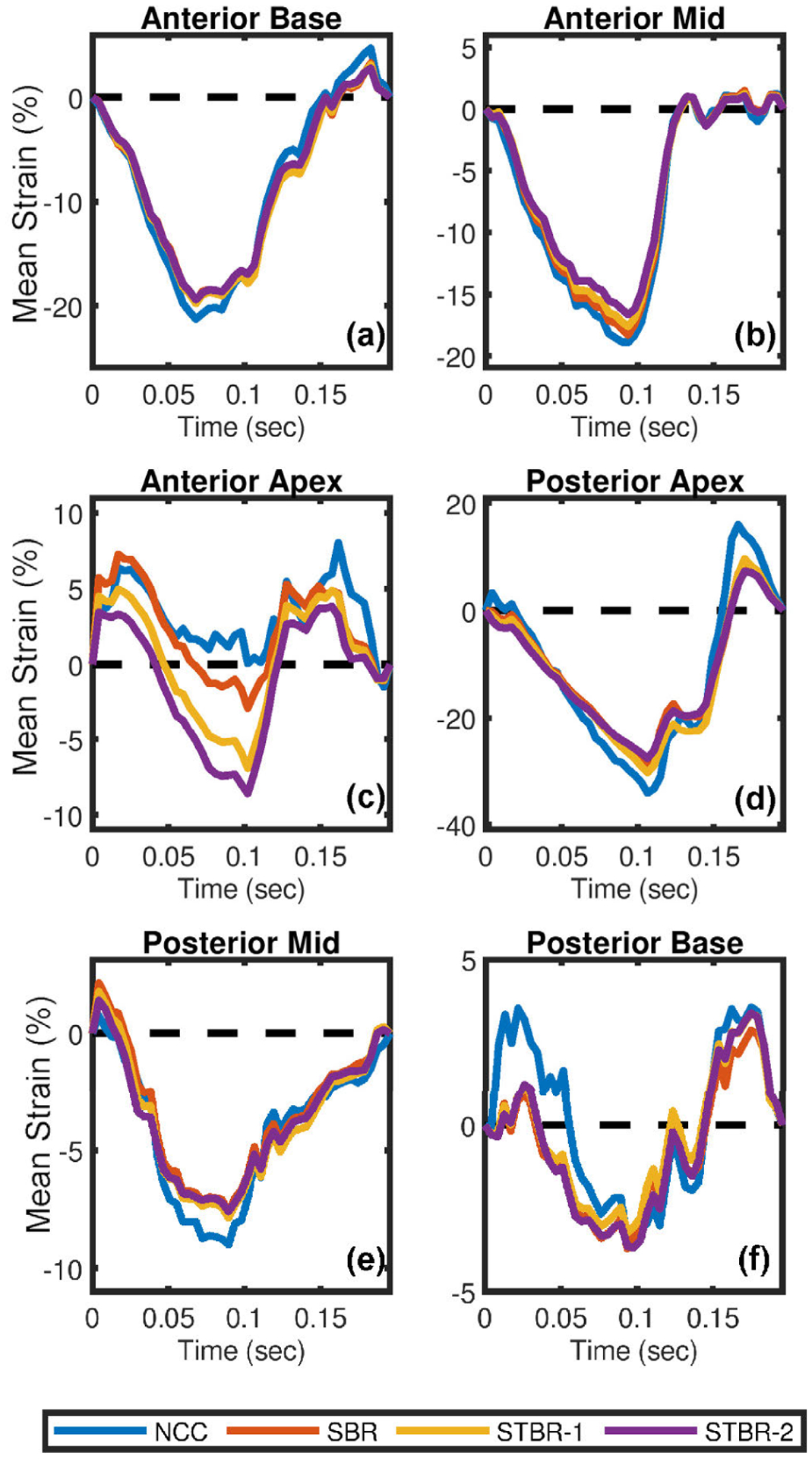
*In vivo* qualitative comparison of longitudinal strain curves. Longitudinal strain curves comparison among NCC, SBR, STBR-1 and STBR-2 for (a) anterior base, (b) anterior mid, (c) anterior apex, (d) posterior apex, (e) posterior mid and (f) posterior base segments respectively.

Figures 14 (a) – (f) qualitatively compare segmental radial strain estimated using NCC, SBR, STBR-1 and STBR-2 for anterior base, anterior mid, anterior apex, posterior apex, posterior mid and posterior base segments respectively. NCC resulted in noisy longitudinal strain curves in the apical [Figure 14 (c)] and posterior base [Figure 14 (f)] segments. SBR provided significant performance improvement in all segments except anterior apex [Figure 14 (c)] with reduced ES longitudinal strain value. STBR-2 had the best quality curves quantified in terms of physiological relevant strain variation and temporal smoothness thus corroborating the ES strain image quality observation from Figure 13.

**FIGURE 15.**
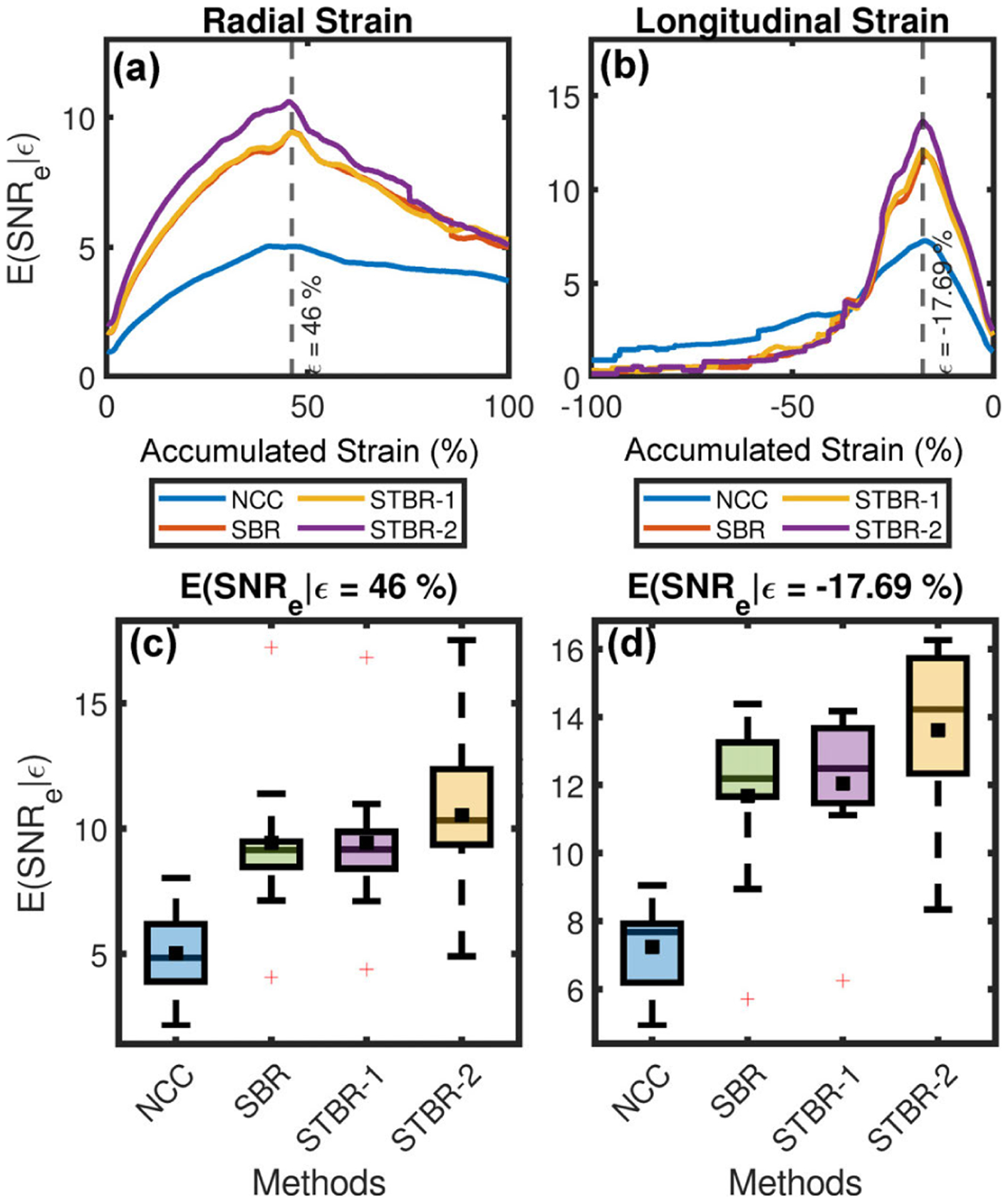
*In vivo* stochastic precision analysis (n = 10). (a) – (b) Radial and longitudinal strain filter comparison, respectively. (c) – (d) Comparison of *E*(SNR_e_ |*ε*) for each method at 46% accumulated radial strain and −17.69% accumulated longitudinal strain, respectively.

Figure 15 summarizes the results for *in vivo* stochastic precision analysis performed using ten healthy mice for radial (Figure 15(a)) and longitudinal (Figure 15(b)) strain filter comparisons, respectively. The strain filter presented in Figure 15 denote the mean of strain filters estimated individually for ten mice. Strain filter comparsion illustrated performance improvement with Bayesian regularization for both radial and longitudinal strain when compared to NCC. SBR and STBR-1 strain filters were coincident with each other indicating no performance improvement with STBR-1. However, STBR-2 produced the strain filters with higher *E*(SNR_e_ |*ε*) values for both *e*_*r*_ and *e*_*l*_ strains. Figures 15 (c) – (d) illustrate the comparison of *E*(SNR_e_ |*ε*) values for each method at 46% accumulated radial strain and −17.69% accumulated longitudinal strain, respectively. All regularization methods performed significantly better than NCC (*p* < *0.05*). Note that STBR-2 had the higher *E*(SNR_e_ |*ε*) values both for radial and longitudinal strains even though it was not statistically significant when compared to SBR and STBR-1. The mean *E*(SNR_e_ |*ε*) values at 46% accumulated radial strain for NCC, SBR, STBR-1 and STBR-2 were 5.03, 9.43, 9.42 and **10.58**, respectively. The mean *E*(SNR_e_ |*ε*) values at −17.69% accumulated longitudinal strain for NCC, SBR, STBR-1 and STBR-2 were 7.24, 11.68, 12.06 and **13.62**, respectively.

**FIGURE 16.**
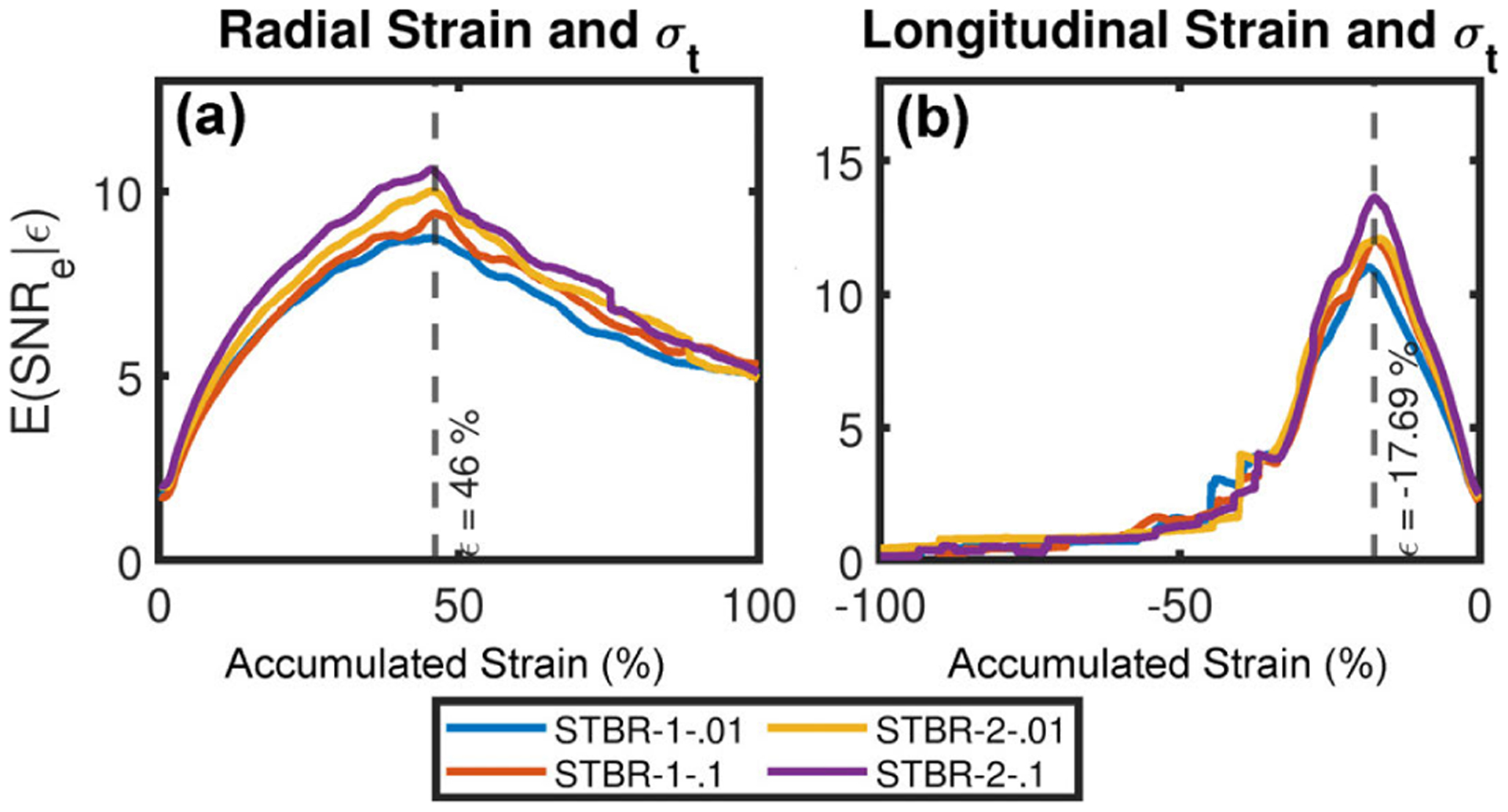
Variation of *in vivo* strain estimation performance as a function of *σ*_*t*_. (a) – (b) Radial and longitudinal strain estimation performance as a function of *σ*_*t*_.

Figures 16 (a) – (b) show the variation of *in vivo* radial strain and longitudinal estimation performance as a function of *σ*_*t*_. For both STBR-1 and STBR-2, we have generated strain filters with ***σ***_**t**_ = [0.01, 0.01] and [0.1, 0.1] respectively. Figure 16 show that ***σ***_**t**_ = [0.1, 0.1] provided higher *E*(SNR_e_ |*ε*) values for both methods with the best performance achieved with STBR-2 for radial and longitudinal strain results. Therefore, width vector = [0.1, 0.1] was used as a default parameter in the *in vivo* study.

Table 2 presents computational times for all methods for inter-frame displacement estimation. The results are measured in seconds and evaluated for a mouse RF dataset. The final RF data dimension was 6016 × 440 and mean execution time for 49 frames covering a complete cardiac cycle is reported. Bayesian methods required more computational time than NCC with highest time required by STBR-1.

## V. DISCUSSION

In this paper, we evaluated two STBR approaches (STBR-1 and STBR-2) and compared them against conventional NCC and spatial Bayesian regularization (SBR) using FEA and *in vivo* small animal studies both qualitatively and quantitatively. The key findings from these studies are summarized as follows.

- Both spatial and spatiotemporal regularization methods performed significantly better than NCC for both FEA simulation and *in vivo* studies.
- For the FEA simulation study, STBR-1 and STBR-2 performed as good as SBR in most of the cases. Few cases resulted in lower estimation errors with STBR however without any statistical significance.
- Incorporation of temporal domain information resulted in better ES strain images and smoother strain curves *in vivo*.
- STBR-2 is the preferred spatiotemporal regularization scheme because of lower errors in FEA simulation and higher SNR_e_ *in vivo*.

Qualitative comparison of ES radial strain images and temporal strain curves derived showed the robustness of Bayesian regularization to handle significant noise corruption when compared to NCC. Posterior segments incurred increased noise artifacts when compared to anterior segments in the FEA simulation because of the modelled frequency dependent acoustic attenuation reducing SNR_s_ with depth. The strain images presented in Figure 3 are reported at the ES phase of cardiac cycle while the average segmental temporal curves shown in Figures 4 represent strain values averaged over the entire cardiac cycle by dividing it into six equal segments. Therefore, the presented ES strain images denote a single time point in the temporal curves. Although the strain images look noisy in the posterior wall, on average the strain values with STBR-1 and STBR-2 were closer to FEA resulting in better qualitative agreement in posterior segments (apex, mid and base) compared to NCC and SBR indicating the benefit of using temporal regularization for low SNR regions [Figs. 3 (c) – (d) and Figs. 4 (d) – (f)]. No statistically significant difference between SBR and STBR methods for higher SNR data was observed. These results suggest that for high SNR input data, additional regularization with temporal information may not be necessary.

**FIGURE 17.**
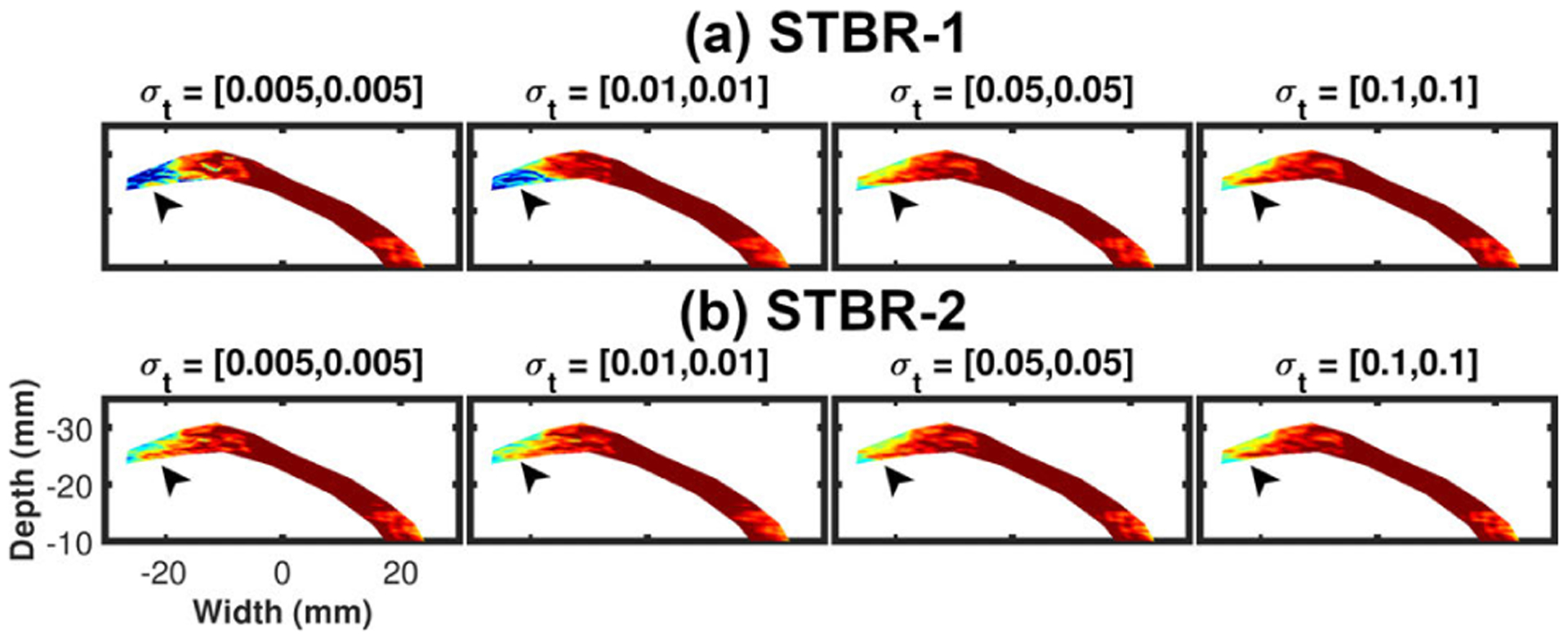
ES radial strain images from (a) STBR-1 and (b) STBR-2 respectively as a function of *σ*_*t*_. STBR-1 shows strain under-estimation in the ROI indicated by arrows (blue pixels) for lower values of *σ*_*t*_. The strain dynamic range is from −10% to +10%.

In addition, spatial then temporal regularization (STBR-1) resulted in under-estimation of radial strain in apical anterior base segment (Figures 3 (d) and 4 (a)) suggesting iterative application of Bayesian regularization with only temporal information might result in undesirable bias due to over-regularization [55]. The prior PDF used in STBR-1 (equation 4) posterior PDF calculation is the posterior PDF calculated after one iteration of spatial Bayesian regularization and the likelihood function only utilizes information from temporal neighbors. Therefore, STBR-1 has the effect of running Bayesian regularization twice (first iteration spatial only and second iteration temporal only) which is not the optimal iteration number for segment 1 (apical base). Furthermore, the chosen width vector value (***σ***_**t**_ [0.01, 0.01]) appeared to be too small thus enforcing higher regularization. On the contrary, STBR-2 uses spatial and temporal information simultaneously resulting in a better safeguard against over-regularization from temporal information only. This can be observed in Figure 17 where STBR-1 showed higher sensitivity towards over-regularization when compared to STBR-2 as a function of width vector values (observe the ROI indicated by arrows).

SBR, STBR-1 and STBR-2 longitudinal results demonstrated good agreement with FEA results compared to NCC with no clear distinction between them [Figures 5 and 6]. These results might be attributed to the simulated higher lateral sampling frequency (500 A-lines) and lateral Sinc interpolation used before displacement estimation [65]. Similar to radial strain images, ES longitudinal strains images from Bayesian methods look noisy in the posterior wall however temporal strain curves showed good agreement due to the averaging over cardiac segments [Figures 5 (c) – (d) and Figures 6 (d) – (f)]. These qualitative findings correlate well with the quantitative evaluation of strain bias, normalized strain error and total temporal relative error. Note that, higher TTRs with STBR-1 compared to SBR and STBR-2 resulted from underestimation with only temporal regularization. Additionally, adaptive application of either SBR or STBR-2 might be a preferred approach for Bayesian regularization depending on local signal decorrelation and input RF data signal-to-noise ratio for future studies.

*In vivo* qualitative results suggest benefits from using temporal information for CSI observed with uniform strain distribution and strain curves with smooth temporal variation and physiological relevance (Figures 11 – 14). Quantitative stochastic analysis results (Figure 15) corroborate the qualitative findings with STBR-2 demonstrating the best performance in terms of *E*(SNR_e_ |*ε*). Even though STBR-2 had higher radial and longitudinal *E*(SNR_e_ |*ε*) values compared to all other methods, the results were not statistically significant possibly due to small sample size (n = 10) and the choice of a conservative post-hoc test (Bonferroni) for multiple comparisons after ANOVA for four algorithms. Additionally, the best performance with STBR-2 correlates with our conclusion from FEA simulation study where STBR-2 is preferred over STBR-1 due to lower errors.

We also demonstrated performance variation with the choice of ***σ***_**t**_ (temporal Gaussian width vector) in FEA simulation and *in vivo* studies [Figures 10 and 16] with optimal ***σ***_**t**_ being 0.01 and 0.1, respectively. One interesting observation from these results is the dependence of ***σ***_**t**_ on image acquisition frame rate (simulation = 250 Hz for canine heart and *in vivo* = 213 Hz mouse heart) suggesting lower ***σ***_**t**_ for data collected at higher frame rate. Our previous *in vivo* STBR for carotid strain imaging also corroborates the finding (optimal ***σ***_**t**_ = 0.005 for carotid artery with imaging frame rate = 538 Hz). ***σ***_**t**_ can be considered as a tuning parameter controlling the type of displacements allowed by the model [note that likelihood function construction in equation (3)]. Lower ***σ***_**t**_ enforce higher temporal continuity and vice versa. Thus, it is reasonable to expect the optimal choice to be tissue and imaging frame rate specific. In this paper, we set ***σ***_**t**_ empirically, a potential drawback which must be addressed before employing STBR for future *in vivo* studies. Possible solutions include dynamic variation of ***σ***_**t**_ based on local signal decorrelation [18], [55], [70] or designing tissue-specific presets for displacement estimation parameters as suggested by Ashikuzzaman *et al*. [16]. Please note that the link between width vector ***σ***_**t**_ and image acquisition frame rate are observational at this point and warrant further investigation in the future by designing appropriate *in vivo* studies.

Computational timing analysis showed that STBR methods require more time to execute when compared to NCC or SBR (Table 2). Additional processing time stems from the referred time loops shown in Figure 2 [Algorithm 1]. Several methods exist to improve computation efficiency. For example, NCC calculation are done within a temporal for loop which calls a CUDA kernel having 2-D blocks of threads. The temporal loop can be replaced with 3-D blocks of threads achieving better parallelization. However, higher memory requirement will be a potential challenge while adopting this approach.

In this work, temporal consistency is designed to be piecewise smooth as information from only immediate past and future neighboring frames is used in contrast to spatiotemporal algorithms which enforce global smooth displacement trajectory over a cardiac cycle [9] with cyclic periodicity [71]. Similar, piecewise temporal smoothness was previously reported in literature for CSI [11], [13]. This constraint is applied to ensure robustness against out-of-plane motion artifacts which can introduce large discontinuity in temporal displacement fields consequently impacting the cardiac strain estimates negatively thus resulting in physiologically more plausible strain variation *in vivo* [observe Figure 12 and 14]. Please note that in case of patients (e.g. arrhythmia) heart movements might be irregular mostly impacting cyclic periodicity [72] however smoothness assumption in a small local neighborhood should still hold based on findings from feasibility studies reported by Elen *et al*. [10]. Moreover, in the preferred spatiotemporal regularization scheme (STBR-2), the spatial neighborhood size is always larger than temporal neighborhood size in our implementation thus ensuring that the algorithm is not biased by temporal information.

Even though we did not observe noticeable performance improvement within our simulation studies, we observed discernable performance improvement with STBR methods (specially SBTR-2) in *in vivo* studies. *In vivo* CSI is significantly challenging due to out-of-plane motion of heart, partially decorrelated speckle pattern, acoustic shadowing and reverberation artifacts from sternum impacting image quality [9]–[11]. We hypothesize that not incorporating these complex imaging conditions in our simulation framework might resulted in the performance difference observed between simulation and *in vivo*. Furthermore, the better performance *in vivo* due to temporal coherence corroborates with findings from previous literature reports [9]–[11], [13], [30].

Several state-of-art US imaging techniques with plane or diverging wave imaging have also been implemented for cardiac and vascular strain imaging applications [34], [73], [74]. These techniques achieve significantly higher frame rates compared to focused line-by-line image acquisition approaches. We anticipate more robust Bayesian regularization for these applications using both spatial and temporal domain information simultaneously.

One limitation of the current study is the use of data only from healthy models for both FEA and *in vivo* studies. To better understand the robustness and efficacy of the STBR, diseased heart models [6] (e.g., ischemia, dyssynchrony, arrhythmia) will be considered in future studies. Another limitation is the algorithm implementation for linear arrays as opposed to phased array transducers. This must be addressed before possible application of STBR to *in vivo* human studies. Finally, our analysis was limited to a single iteration of temporal regularization thus sampling information only from its immediate past and future neighbors. In our previous work on Bayesian regularization and CSI [31], [55], we have found that a single iteration was sufficient for optimal performance in the FEA simulation model. For consistency between simulation and *in vivo* studies, we set the iteration number to be one in our *in vivo* study. With higher iteration number, information beyond adjacent spatial and temporal neighbors will be incorporated in STBR which may lead to over-regularization if the algorithm is not adaptive [55]. Iterative application will be investigated in future studies to better understand the effect of neighborhood size for STBR.

## VI. CONCLUSION

Spatiotemporal Bayesian regularization is applied to a Lagrangian cardiac strain estimation framework in this paper. The proposed algorithm was validated using a FEA canine deformation model and *in vivo* healthy murine data sets. Our results suggest that Bayesian regularization benefits with additional temporal information specially when applied *in vivo*.

## Supplementary Material

supp1-3130021

## Figures and Tables

**TABLE 1.** Displacement estimation parameters for FEA simulation and in vivo studies.

	Value	Unit
Number of levels	3	-
RF data sampling factor [Axial: Lateral]	1:2	-
Axial decimation factors	[3,2,1]	-
Lateral decimation factors	[2,1,1]	-
Axial kernel length	[8*λ*, 5*λ*, 1*λ*]	Wavelengths
Lateral kernel length	[15, 12, 10]	A-lines
Kernel overlaps [Axial, Lateral]	[10*,90]	%
Median filter kernel [Axial, Lateral]	[5** × 5]	pixels
Subsample estimation	2-D Sine	-

* *In vivo* axial kernel overlap was 50 %

** *In vivo* median filter axial kernel dimension was 7 pixels

**TABLE 2.** Summary of computational time (seconds).

NCC	SBR	STBR-1	STBR-2
73.20	114.30	316.15	156.86
