# Supplementary material for: Spatiotemporal Bayesian Regularization for Cardiac Strain Imaging: Simulation and In Vivo Results
Source: IEEE Open J Ultrason Ferroelectr Freq Control. Author manuscript; Available in PMC 2022 Feb 15. (PMC8846604; doi:10.1109/OJUFFC.2021.3130021)
Supplement: supp1-3130021 [file NIHMS1764436-supplement-supp1-3130021.pdf]

Supplemental Material for "Spatiotemporal Bayesian Regularization for Cardiac Strain Imaging: Simulation and *In vivo* Results"

Displacement Analysis Results for FEA Canine Model

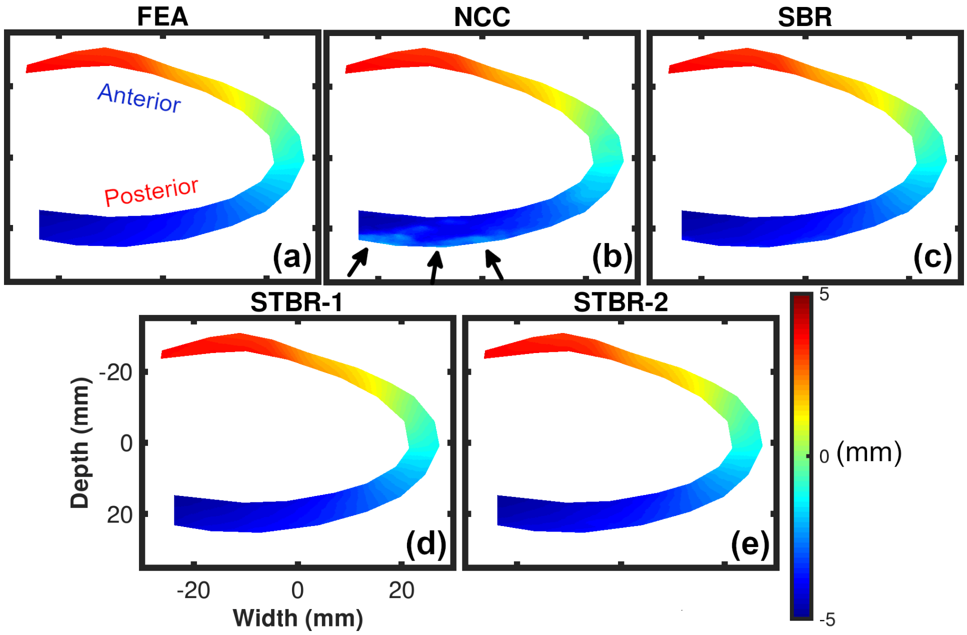

Figure 1: **Qualitative comparison of end-systole (ES) cumulative axial displacement estimation for FEA simulations.** (a) – (e) denote FEA, NCC, SBR, STBR-1 and STBR-2 results, respectively. SBR = Spatial Bayesian regularization, STBR-1 = Spatial then temporal Bayesian regularization and STBR-2 = Simultaneous STBR.

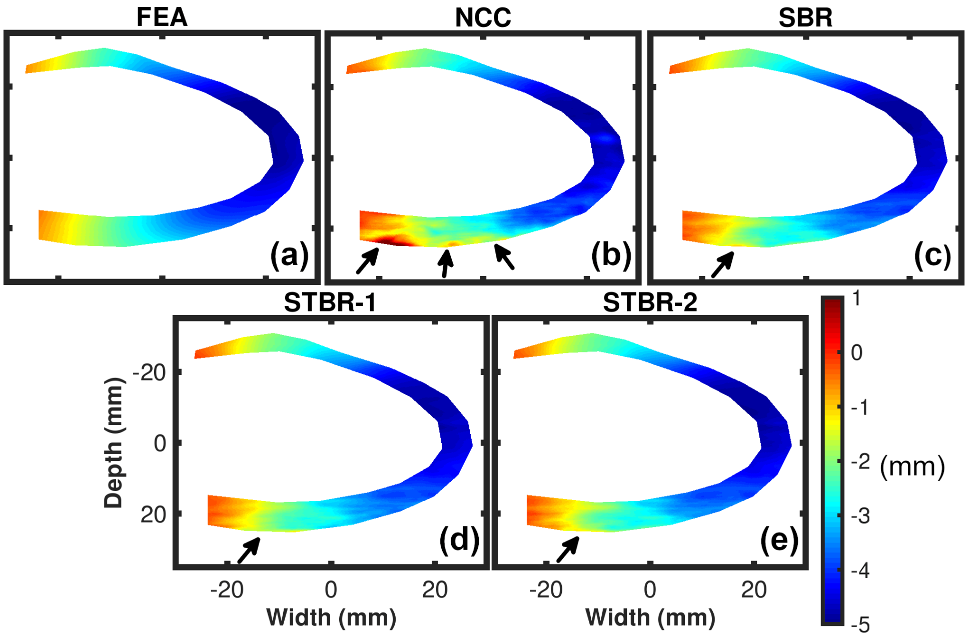

Figure 2: **Qualitative comparison of end-systole (ES) cumulative lateral displacement estimation for FEA simulations.** (a) – (e) denote FEA, NCC, SBR, STBR-1 and STBR-2 results, respectively. SBR = Spatial Bayesian regularization, STBR-1 = Spatial then temporal Bayesian regularization and STBR-2 = Simultaneous STBR.

Figures 1 (a) – (e) show end-systole (ES) cumulative axial displacement images obtained using FEA model, NCC, SBR, STBR-1 and STBR-2, respectively. Input RF data for this example had SNR_s value of 15 dB at anterior wall and 0 dB at posterior wall. NCC images deviated from FEA result in the posterior wall indicated by black arrows. SBR, STBR-1 and STBR-2 demonstrated excellent agreement with FEA with no easily observable difference among each other.

In a similar manner, Figures 2 (a) – (e) show ES cumulative lateral displacement images obtained using FEA model, NCC, SBR, STBR-1 and STBR-2, respectively. NCC images deviated from FEA result in the posterior wall indicated by black arrows. STBR-1 and STBR-2 demonstrated slightly better performance compared to SBR at posterior wall indicated by the arrows with no easily observable difference among each other.

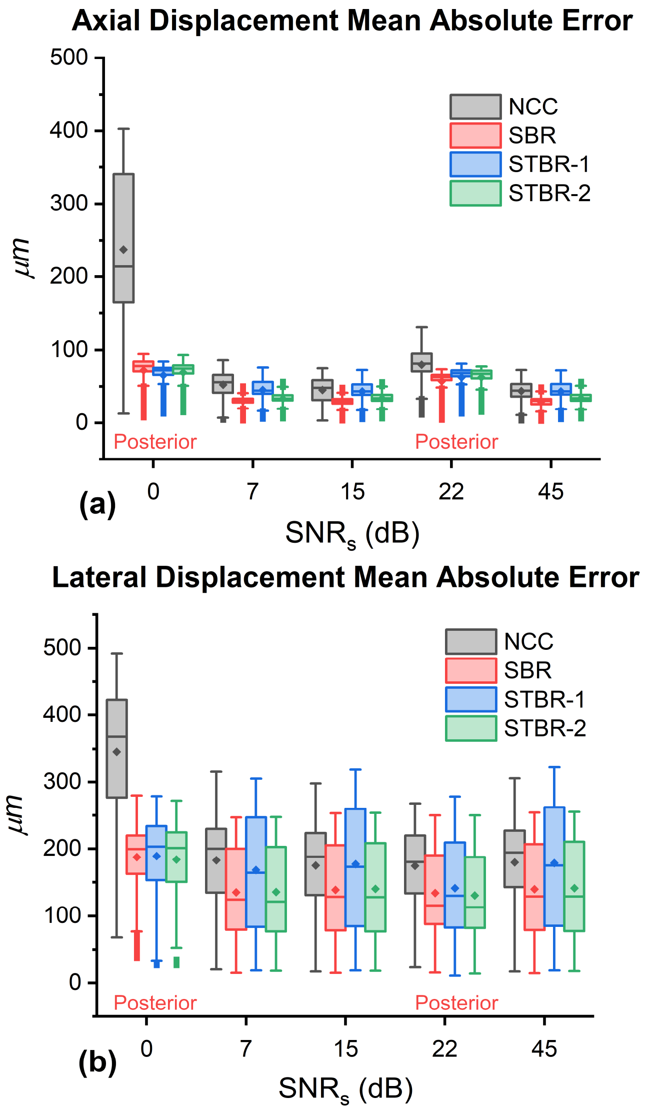

Figure 3: **Displacement mean absolute error (MAE) comparison (n = 620).** (a) – (b) Cumulative axial and lateral displacement MAE comparison results respectively as a function of SNR_s levels sampled from anterior and posterior walls. SNR_s = 0 and 22 dB were from posterior wall.

Figures 3 (a) – (b) shows quantitative comparison results of cumulative axial and lateral displacement mean absolute error comparison results respectively as a function of SNR_s sampled from anterior and posterior walls. Mean absolute error (MAE) was computed using the following equation.

$$MAE = \frac{\sum_{i=1}^{P} \left| d_{true} - d_{est} \right|}{P} \quad (1)$$

where, d_{true}, d_{est} and P denote FEA displacement, estimated displacement and number of points in the cardiac mesh (24000 points) respectively.

Figure 3 (a) shows that the Bayesian methods (SBR, STBR-1 and STBR-2) outperform NCC with lower MAE specially in the posterior segments (please refer to Figure 1 (a) to identify posterior wall). SBR and STBR-2 performed better than STBR-1 specially in the anterior segments corroborating our conclusion of STBR-2 being the preferred spatiotemporal regularization method. Similar results were seen for cumulative lateral displacement (Figure 3 (b)). SBR and STBR-2 had lower mean MAE than STBR-1 specially in the anterior segments and all Bayesian methods outperformed NCC showing the benefit of regularization for lateral displacement estimation.
